# Elucidating the modes of action for bioactive compounds in a cell-specific manner by large-scale chemically-induced transcriptomics

**DOI:** 10.1038/srep40164

**Published:** 2017-01-10

**Authors:** Michio Iwata, Ryusuke Sawada, Hiroaki Iwata, Masaaki Kotera, Yoshihiro Yamanishi

**Affiliations:** 1 Division of System Cohort, Medical Institute of Bioregulation, Kyushu University, 3-1-1 Maidashi, Higashi-ku, Fukuoka, Fukuoka 812-8582, Japan; 2 School of Life Science and Technology, Tokyo Institute of Technology, 2-12-1 Ookayama, Meguro-ku, Tokyo, 152-8550, Japan; 3 Institute for Advanced Study, Kyushu University, 3-1-1 Maidashi, Higashi-ku, Fukuoka, Fukuoka 812-8582, Japan; 4 PRESTO, Japan Science and Technology Agency, Kawaguchi, Saitama 332-0012, Japan

## Abstract

The identification of the modes of action of bioactive compounds is a major challenge in chemical systems biology of diseases. Genome-wide expression profiling of transcriptional responses to compound treatment for human cell lines is a promising unbiased approach for the mode-of-action analysis. Here we developed a novel approach to elucidate the modes of action of bioactive compounds in a cell-specific manner using large-scale chemically-induced transcriptome data acquired from the Library of Integrated Network-based Cellular Signatures (LINCS), and analyzed 16,268 compounds and 68 human cell lines. First, we performed pathway enrichment analyses of regulated genes to reveal active pathways among 163 biological pathways. Next, we explored potential target proteins (including primary targets and off-targets) with cell-specific transcriptional similarity using chemical–protein interactome. Finally, we predicted new therapeutic indications for 461 diseases based on the target proteins. We showed the usefulness of the proposed approach in terms of prediction coverage, interpretation, and large-scale applicability, and validated the new prediction results experimentally by an *in vitro* cellular assay. The approach has a high potential for advancing drug discovery and repositioning.

The identification of the modes of action of bioactive compounds is a major challenge in chemical systems biology of diseases. Diseases are caused by dysfunction of the human biological system, which consists of genes, proteins, and pathways. Most drugs are bioactive compounds that modulate the activity of biological systems for the treatment of diseases. However, there are numerous bioactive compounds (including approved drugs) whose mechanisms are unknown. Drugs interact with target proteins implicated in a disease of interest and are indispensable for maintaining human physiology. However, drugs may interact not only with their primary target proteins but also with other proteins (off-targets) and thereby cause unexpected side effects^1^,^2^,^3^. Side effects derived from off-target interactions, although typically undesired, may occasionally enable new therapeutic indications^4^,^5^,^6^ because different diseases often share same or similar pathogenic mechanisms. Therefore, understanding the complex responses of the human biological system to bioactive compounds is of vital importance in medical and pharmaceutical research.

Genome-wide expression profiling of transcriptional responses to compound treatment for human cell lines is a promising unbiased approach for exploring the modes of action of bioactive compounds. In recent years, chemically-induced gene expression data have become available via several public databases. A toxicogenomics project was conducted in Japan between 2002 and 2006, and the results are stored in the Toxicogenomics Project-Genomics Assisted Toxicity Evaluation system (TG-GATEs), where 170 compounds were used to perturb cell homeostasis *in vitro*^7^. In 2006, the Connectivity Map (CMap) database was constructed in the United States, storing the gene expression profiles of five cancer cell lines perturbed by 1,309 compounds^8^. These databases enable to relate compound activities with gene expression patterns.

A variety of pharmaceutical studies have been performed based on the transcriptome resources. A popular approach for drug repositioning based on CMap is to search for compounds whose gene expression patterns are inversely correlated with those of a disease of interest^9^,^10^,^11^. CMap data have been analyzed to evaluate the transcriptional similarity of drugs^12^, and the transcriptional similarity has been used to predict the targets of drugs^13^,^14^. A module detection approach was used in efforts to detect drug-induced transcriptional modules that were conserved among different cell lines, as well as among different organisms^15^, and a probabilistic approach was proposed for the information retrieval problem in CMap^16^. Normalization methods and gene selection procedures have been proposed to handle the batch effect problems in CMap^17^,^18^,^19^,^20^. However, the characteristics of cell-specific gene expression profiles and differences in gene expression profiles between cell lines have not been taken into account in these previous studies. In addition, the previous methods heavily depended on the coverage of drugs in CMap and the availability of gene expression data for diseases, which limit large-scale analyses.

Recently, the Library of Integrated Network-based Cellular Signatures (LINCS) L1000 data were established by the Broad Institute in the United States^21^. The LINCS database stores a huge number of gene expression profiles that include the transcriptomic responses of 77 human cell lines to various perturbations, including compound treatment, gene knockdown, gene overexpression, and several more interventions. For compound treatment, gene expression profiles are available for 20,413 compounds and 72 human cell lines, which are much larger than those available from CMap. Thus, the LINCS resources should be valuable for medical and pharmaceutical research, and there are some recent reports on the use of LINCS. For example, a normalization method specific to raw data within LINCS L1000 data was proposed^22^, and a large-scale correlation analysis of chemical structures and gene expression profiles was performed^23^. There is therefore a strong incentive to develop computational methods to effectively use LINCS for a variety of pharmaceutical applications.

In this study, we developed a novel approach to elucidate the modes of action of bioactive compounds in a cell-specific manner using large-scale chemically-induced transcriptome data acquired from LINCS. We applied the proposed approach to 16,268 compounds and 68 human cell lines, which enabled the comprehensive prediction of active pathways, target proteins, and therapeutic indications of the compounds. We showed the usefulness of the proposed approach in terms of prediction coverage, interpretation, and large-scale applicability, and validated the new prediction results experimentally by an *in vitro* cellular assay. The approach has a high potential for advancing drug discovery and repositioning.

## Results

### LINCS provides large-scale transcriptome data for compound activity

We compared the statistics of the chemically-induced gene expression profile data contained within three different databases: TG-GATEs, CMap, and LINCS. Supplementary Fig. S1 shows a Venn diagram of the numbers of cell lines and compounds included in the three databases. In LINCS, we focused on gene expression data measured at 6 h after compound treatment. The numbers of cell lines included in TG-GATEs, CMap, and LINCS were 1, 5, and 68, respectively. Three human cancer cell lines (MCF7, PC3, and HL60) were used in both CMap and LINCS, and one primary hepatocyte was used only in TG-GATEs. The numbers of compounds included in TG-GATEs, CMap, and LINCS were 157, 1,133, and 16,268, respectively. Approximately 500 compounds were used in both CMap and LINCS. The numbers of cell lines and compounds used in LINCS were much larger than those used in TG-GATEs and CMap. This suggests the potential of LINCS for large-scale applications.

**Figure 1:**
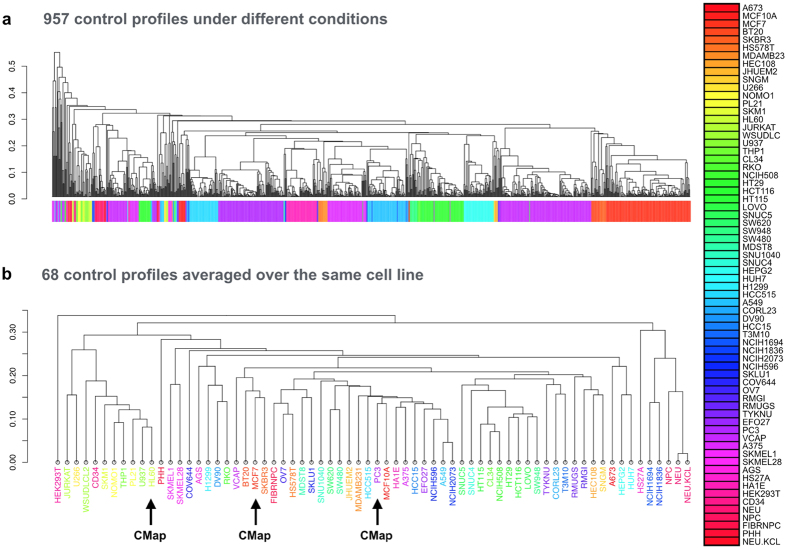
Clustering of cell lines using transcriptional similarity scores between control profiles in LINCS. Individual cell lines are indicated by different colors, and cell lines obtained from the same tissue are indicted by similar colors. Dendrogram (**a**) shows the result of clustering 957 control profiles. Dendrogram (**b**) shows the result of clustering 68 cell lines, where multiple control profiles are averaged over the same cell line. Black arrows indicate cell lines also present in CMap.

We analyzed control gene expression profiles, measured at 6 (6.4) h, of 68 human cell lines in LINCS by performing hierarchical clustering. Figure 1a shows the clustering dendrogram of 957 control gene expression profiles, where multiple control profiles under different conditions were prepared for each human cell line. In contrast, Fig. 1b shows the clustering dendrogram of 68 control gene expression profiles, where multiple control gene expression profiles were averaged into a single profile for each of 68 human cell lines. It can be seen that the same cell lines and similar cell lines from the same tissues tend to be closely located, while the cell lines from different tissues tend to be scattered and distantly located. For example, the cell lines used in CMap (i.e., MCF7, PC3, and HL60) were scattered and distantly located as well. These results suggest that gene expression profiles are highly variable among different cell lines and that it important to take into account cell-specific gene expression profiles.

### An overview of the proposed approach

**Figure 2:**
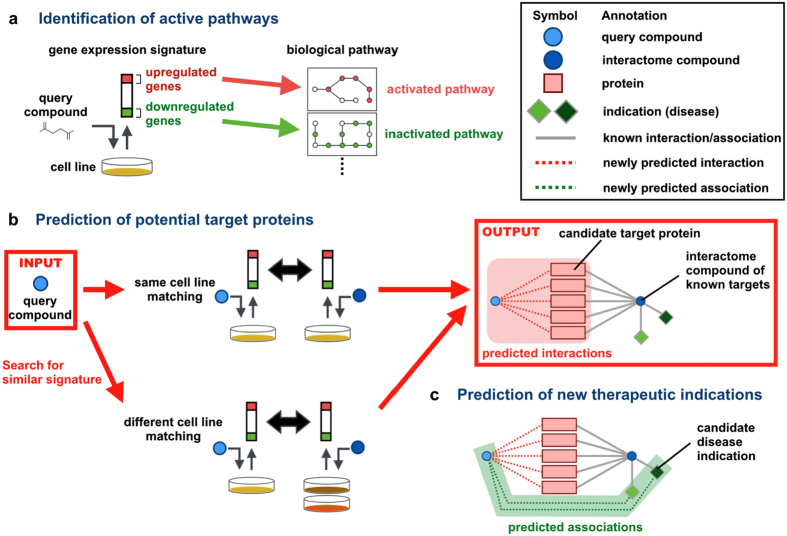
Overview of the proposed approach. (**a**) Identification of active pathways, (**b**) prediction of potential target proteins, and (**c**) prediction of new therapeutic indications.

We analyzed a set of chemically-induced gene expression profiles (referred to as “gene expression signatures” or “signatures” for short) in which each element of the signature is the logarithmic ratio of the gene expression measured after compound treatment to that measured in the control condition. Here, we present an overview of the proposed approach to elucidate the mode of action of a given bioactive compound based on its gene expression signature. The proposed approach consists of three steps: (i) identification of active pathways, (ii) prediction of potential target proteins, and (iii) prediction of new therapeutic indications. Figure 2 illustrates this approach.

Figure 2a shows the procedure for identifying biological pathways in which the genes are activated or inactivated by a query compound. The upregulated and downregulated genes in the signatures are mapped onto many biological pathway maps, and the statistical significance of the enrichment of the regulated genes in each pathway is evaluated using a hypergeometric test.

Figure 2b shows the procedure for predicting potential target proteins of a query compound. Transcriptional similarities are evaluated by calculating correlation coefficients between gene expression signatures in a cell-specific manner. A set of compounds with known target proteins, called “interactome compounds,” is prepared in advance. If the query compound is transcriptionally similar to a set of interactome compounds, the query compound is predicted to have the same target proteins as the most similar compound.

Figure 2c shows the procedure for predicting new therapeutic indications of a query compound. If the query compound is transcriptionally similar to a set of interactome compounds with known indications and shares the same target proteins with these interactome compounds, the query compound is predicted to have the same therapeutic indications as the most similar compound.

### Identification of activated and inactivated pathways

**Figure 3:**
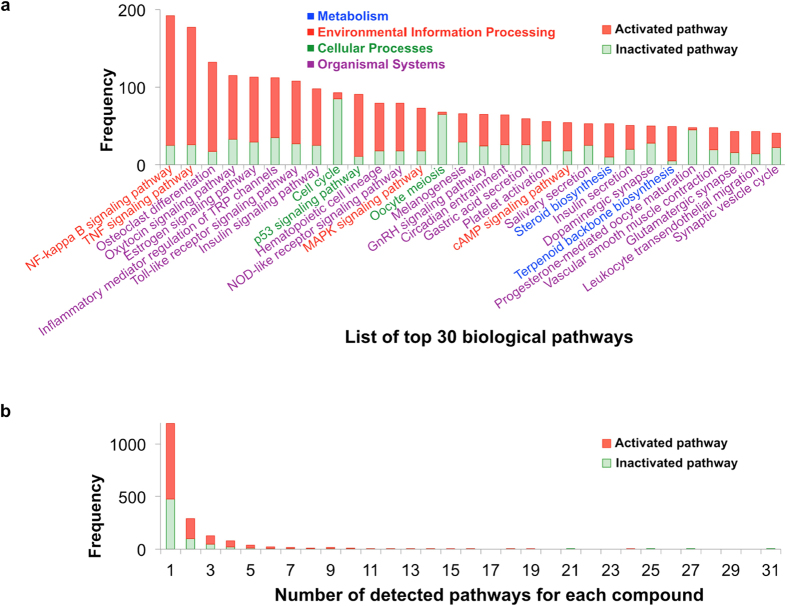
Distribution of the identified pathways. (**a**) The histogram of detected pathways by the result of analyzing all compounds, where the horizontal axis indicates the list of biological pathways and the vertical axis indicates the frequency of detected pathways. (**b**) The histogram of the numbers of detected pathways for each compound, where the horizontal axis indicates the number of detected pathways for each compound and the vertical axis indicates the frequency of compounds. Red bars indicate the numbers of activated pathways, identified using upregulated genes, and green bars indicate the numbers of inactivated pathways, identified using downregulated genes. The pathways found in the KEGG categories Metabolism, Environmental Information Processing, Cellular Processes, and Organismal Systems are colored blue, red, green, and purple, respectively.

Pathway enrichment analyses were performed on 16,268 bioactive compounds, and their pathway activities for 163 biological pathways in the Kyoto Encyclopedia of Genes and Genomes (KEGG) database^24^ were inferred. Figure 3a shows the distribution of pathways detected at a statistically significant level (*P* < 0.05) for all compounds. Our analysis detected 119 unique pathways, but showed only top 30 pathways because of space limitation. Genes in a variety of pathways were activated or inactivated by the chemical perturbations. For example, genes in the NF-*κ*B signaling pathway (hsa04064) were frequently activated, and genes in the cell cycle pathway (hsa04110) were frequently inactivated. Genes in the NF-*κ*B signaling pathway were activated by many drugs, such as triamterene (D00386; a diuretic agent) and disulfiram (D00131; an alcohol deterrent agent); these activations were confirmed experimentally in ChEMBL assay 1613823. Many genes in the cell cycle pathway (hsa04110) (as shown in Supplementary Fig. S2) were downregulated upon treatment with anti-cancer drugs, such as the drugs classified as “code L: anti-neoplastic and immunomodulating agents” in the Anatomical Therapeutic Chemical classification system (ATC code), and it is known that most anti-cancer agents interfere with cell proliferation by inhibiting the cell cycle machinery. However, terfenadine (D00521), which is a histamine H1 receptor antagonist, not an anti-cancer drug, significantly inhibits the growth of human cancer cells by inducing G(0)/G(1) phase cell-cycle arrest^25^. These observations indicate that our pathway enrichment analysis results are reasonable.

Figure 3b shows the distribution of the numbers of pathways in which the genes were frequently activated or inactivated by each compound. Genes upregulated or downregulated by most compounds were significantly enriched in a limited number of pathways. This result implies that compounds acting on the same pathway may be good candidates for drug repositioning.

**Figure 4:**
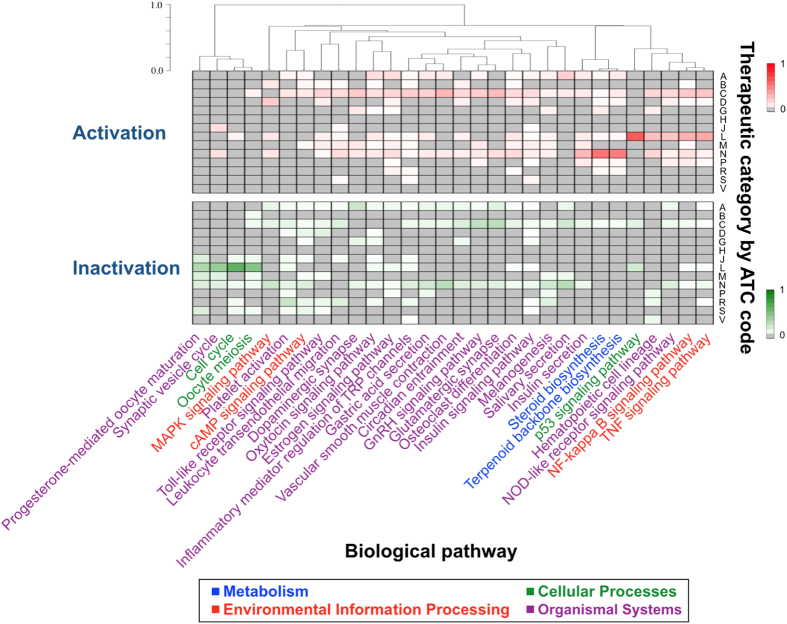
Distribution of drug classifications according to the biological pathways that they activate (top) and inactivate (bottom). The dendrogram shows the result of clustering pathways according to their similarities of the drug classifications. The fraction of drugs in a particular classification that affect each pathway is represented by the intensity of color in the appropriate box. The boxes are arranged according to the first level of the Anatomical Therapeutic Chemical classification system (ATC code). Drugs are assigned the following ATC codes: code A: alimentary tract and metabolism; code B: blood and blood-forming organs; code C: cardiovascular system; code D: dermatologicals; code G: genitourinary system and sex hormones; code H: systemic hormonal preparations, excluding sex hormones and insulins; code J: anti-infectives for systemic use; code L: anti-neoplastic and immunomodulating agents; code M: musculo-skeletal system; code N: nervous system; code P: anti-parasitic products, insecticides and repellents; code R: respiratory system; code S: sensory organs; and code V: various.

Next, we examined the relationship between the detected pathways and drug therapeutic categories, where drugs are a subset of all compounds in our dataset. Figure 4 shows the distribution of the drug classes on the basis of the first level of ATC code in the top-ranked detected pathways, and the detailed explanations on the ATC codes are shown in the figure caption. It was observed that some pairs of the detected pathways were highly associated with similarly classified drugs. For example, the genes in the steroid biosynthesis pathway (hsa00100) and those in the terpenoid backbone biosynthesis pathway (hsa00900) are regulated by similar drugs (ATC codes: A, C, D, L, N, P, and R); in particular, neurological agents tend to activate these pathways. Steroids in humans are biologically synthesized from terpenoid precursors, which suggests the validity of the similarity. The genes in the cell cycle pathway (hsa04110) and those in the oocyte meiosis pathway (hsa04114) are inactivated by similar drugs (ATC codes: J, L, N, and S). This result is reasonable because the two biological pathways are known to function similarly, to some extent. Also, it was observed that anti-neoplastic agents (ATC code: L) tend to activate the NF-*κ*B (hsa04064), TNF (hsa04668), and p53 signaling pathways (hsa04115) and inactivate the cell cycle pathway (hsa04110) and synaptic vesicle cycle pathways (hsa04721). The observations are reasonable from the viewpoint of the modes of action of anti-cancer drugs because the inhibition of cell growth and activation of tumor-suppressor genes, such as *p53*, are beneficial for the treatment of cancers. These results imply that the pathway enrichment analysis provides some clues for understanding the modes of action of bioactive compounds at the pathway level.

The number of compounds (drugs) are different between ATC codes. Compounds are not evenly distributed in the ATC codes in the original dataset published by WHO. For the bias problem, we attempted to use relative frequency (not absolute frequency by simply counting) for comparison between different ATC codes. The corresponding result is shown in Supplementary Fig. S3, where color intensity indicates the relative frequency (the compound frequency was divided by the number of compounds in each ATC code). The same tendency was observed.

We performed the same analysis based on not only the KEGG Pathway database but also the REACTOME Pathway database^26^. We summarized the corresponding pathway enrichment analysis results in Supplementary Figs S4, S5 and S6. The same tendency was observed.

### Performance evaluation of target protein prediction

To test the ability of the proposed method to predict the target proteins of compounds, we performed the following fivefold cross-validation experiment using known compound–target interactions as gold standard data (see Methods). First, we randomly split the compounds in the gold standard data set into five subsets of roughly equal size. Second, we took each subset of compounds as test compounds and regarded the associated compound–target pairs as a test set. Third, we used the remaining four subsets of compounds as training compounds and regarded the associated compound–target pairs as a training set. Finally, we built a predictive model based on only the training set and used it to predict the targets of the compounds in the test set. This process was repeated four more times, each time using a different subset as the test set. We then evaluated the prediction accuracy based on the prediction scores of test compound–target pairs over the five separate experiments. Note that only the compounds were split into training and test sets, and target proteins were common.

We evaluated prediction performance using the receiver operating characteristic (ROC) curve, which is a plot of true–positive rates as a function of false–positive rates, and the precision–recall (PR) curve, which is a plot of precision (positive predictive value) as a function of recall (sensitivity). We summarized performance using the area under the ROC curve (AUC) score, where 1 is perfect inference and 0.5 is random inference, and the area under the PR curve (AUPR) score, where 1 is perfect inference and the ratio of positive examples in the gold standard data is random inference.

Table 1a shows the AUC and AUPR scores for the common data, obtained in the cross-validation experiment where the common data consisted of 391 compounds. We used the genes (L1000 genes) for the comparison between CMap and LINCS. The AUC and AUPR scores obtained using LINCS were slightly lower or almost the same as those obtained using CMap. These results suggest that the quality of the gene expression data present in LINCS is almost the same as that for the data present in CMap for the same compounds.

Table 1b shows the AUC and AUPR scores for the merged data, obtained in the cross-validation experiment, where the merged data consist of 3,767 compounds and the prediction scores of compounds for which gene expression data were unavailable were set to zero. The AUC and AUPR scores obtained using LINCS were much higher than those obtained using CMap. These results suggest that the higher coverage of gene expression data in LINCS leads to better performance for compound target prediction.

We compared the performance of similarity searches performed using two different strategies. The “same cell line-matching” strategy worked better than the “different cell line-matching” strategy. This implies that chemically-induced gene expression has cell-specific features as well as features common to all cells. The integration of the two matching strategies did not make a difference, as shown in Supplementary Tables S1 and S2. Thus, we applied the same cell line-matching strategy for further analyses.

To examine the robustness of the results, we tested the use of different data preprocessing procedures in the construction of gene expression signatures. For example, we compared the performance of two normalization procedures used to construct gene expression profiles: the “biological control” normalization procedure^8^ and the “mean centering” normalization procedure^17^. The biological control normalization procedure worked slightly better than the mean centering normalization procedure in the both cases (Table 1). Thus, we used the gene expression signatures constructed using the biological control normalization procedure in the following experiments.

Next, we tested the top to bottom gene selection procedure using only genes displaying large changes in expression^12^,^13^,^17^,^18^,^19^,^20^, but were not able to confirm that there were significant differences between the “top50”,“top100”, and “all” results (Supplementary Tables S1 and S2). We also examined the effect of using different time points after compound treatment (6 h and 24 h) to assess gene expression, but there was no significant difference in the performance of target protein prediction, as shown in Supplementary Tables S1 and S2. Thus, we used expression data for all genes obtained 6 h after compound treatment in the following analyses.

We performed the same analysis based on tissue types as well as cell lines. We constructed compound-induced gene expression profiles for each tissue type, where cell lines from the same tissue are averaged. We summarized the corresponding cross-validation results for target prediction in Supplementary Table S3. The same tendency was observed. There was little difference of accuracy between cell lines and tissue types.

### Large-scale prediction of potential target proteins and new therapeutic indications

We made a comprehensive prediction of unknown target proteins of 16,268 compounds for which gene expression data were available in LINCS. We used all known compound–protein interactions as learning data and predicted new compound–protein interactions. Supplementary Fig. S7 shows a Venn diagram of the overlap of the results between LINCS-based prediction and CMap-based prediction, where the upper 5th percentile of the prediction score distribution was used as a threshold. The LINCS-based prediction with the same cell line-matching strategy provided 1,776,734 new compound–protein interactions (involving 7,007 compounds and 2,275 proteins), while the CMap-based prediction provided 82,119 new compound–protein interactions (involving 655 compounds and 1,318 proteins). The LINCS-based method provided a larger number of newly predicted compound–protein pairs than the CMap-based method, which was expected because LINCS covers a larger number of cell lines and compounds (Supplementary Fig. S1).

Next, we made a comprehensive prediction of unknown therapeutic indications of 16,268 compounds based on the results of predicted compound targets. Supplementary Fig. S8 shows a Venn diagram of the overlap of the results obtained using LINCS-based and CMap-based predictions. The LINCS-based prediction with the same cell line-matching strategy provided 76,788 new compound–disease associations (involving 4,146 compounds and 250 diseases), while the CMap-based prediction provided 3,340 new compound–disease associations (involving 361 compounds and 191 diseases). The LINCS-based prediction provided a much larger number of new compound–disease pairs than the CMap-based prediction.

**Figure 5:**
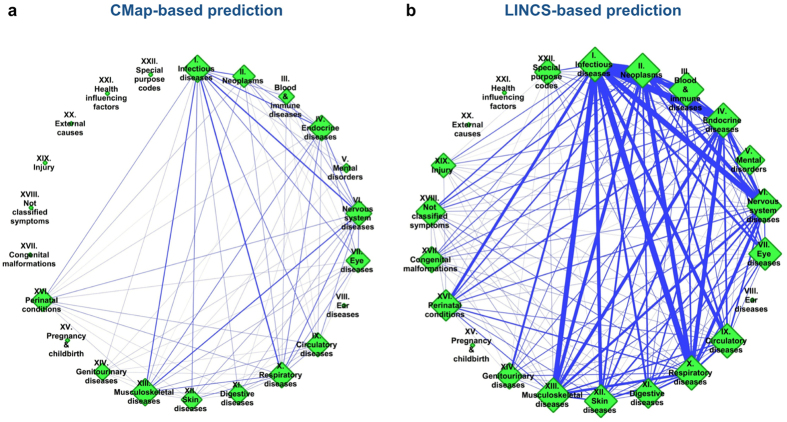
Distribution of drugs repositioned from the original disease class to other disease classes using transcriptional similarity based on CMap (**a**) and LINCS (**b**). Nodes (green diamonds) represent the ICD disease chapters; they are shown with chapter number and short chapter name. Edges (blue lines) indicate potential correlations between diseases according to the transcriptional similarity of drugs. Node size indicates the sum of the edges of each node. Edge width indicates the number of drugs repositioned between two disease chapters. Chapter I: certain infectious and parasitic diseases (A00–B99); chapter II: neoplasms (C00–D48); chapter III: diseases of the blood, blood-forming organs, and certain disorders involving the immune mechanism (D50–D89); chapter IV: endocrine, nutritional, and metabolic diseases (E00–E90); chapter V: mental and behavioral disorders (F00–F99); chapter VI: diseases of the nervous system (G00–G99); chapter VII: diseases of the eye and adnexa (H00–H59); chapter VIII: diseases of the ear and mastoid process (H60–H95); chapter IX: diseases of the circulatory system (I00–I99); chapter X: diseases of the respiratory system (J00–J99); chapter XI: diseases of the digestive system (K00–K93); chapter XII: diseases of the skin and subcutaneous tissue (L00–L99); chapter XIII: diseases of the musculoskeletal system and connective tissue (M00–M99); chapter XIV: diseases of the genitourinary system (N00–N99); chapter XV: pregnancy, childbirth, and the puerperium (O00–O99); chapter XVI: certain conditions originating in the perinatal period (P00–P96); chapter XVII: congenital malformations, deformations; and chromosomal abnormalities (Q00–Q99); chapter XVIII: symptoms, signs, and abnormal clinical and laboratory findings not elsewhere classified (R00–R99); chapter XIX: injury, poisoning, and certain other consequences of external causes (S00–T98); chapter XX: external causes of morbidity and mortality (V01–Y98); chapter XXI: factors influencing health status and contact with health services (Z00–Z99); and chapter XXII: codes for special purposes (U00–U99).

We examined the results of the prediction for approved drugs, which was a subset of the compounds used in this study. Figure 5 shows the distributions of drugs repositioned from the original disease class to other disease classes based on compound transcriptional similarity, where diseases were classified using the ICD-10 disease classes, referred to as “ICD chapter”^27^. The detailed numbers of repositioned drugs are shown in Supplementary Tables S4 and S5, respectively. Figures 5a and b show the distribution of repositioned drugs between different ICD chapters using the CMap-based and the LINCS-based predictions, respectively. The CMap-based prediction resulted in the largest number of drugs possibly repositioned from chapter I (certain infectious and parasitic diseases) to chapter VI (diseases of the nervous system) and *vice versa*, followed by the possible drug repositioning from chapter I (certain infectious and parasitic diseases) to chapter X (diseases of the respiratory system) and *vice versa*. In contrast, the LINCS-based prediction resulted in the largest number of drugs possibly repositioned from chapter I (certain infectious and parasitic diseases) to chapter II (neoplasms) and *vice versa*, followed by the possible drug repositioning from chapter I (certain infectious and parasitic diseases) to chapter IV (endocrine, nutritional, and metabolic diseases) and *vice versa*. These are not the only differences between the CMap-based and LINCS-based prediction results. As clearly shown in Fig. 5, the LINCS-based prediction provided potential new therapeutic indications for a wider range of diseases, which may result in practical applications for drug repositioning. Thus, we analyzed the results obtained using only the LINCS-based method below.

### Biological interpretations of the drug–protein–disease network

**Figure 6:**
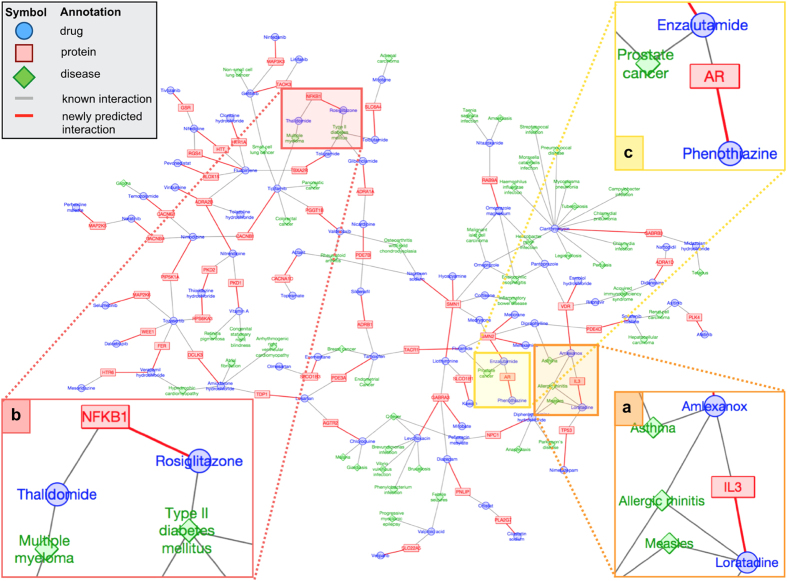
A small part of the drug–protein–disease network predicted using the LINCS-based method. Three panels denoted to as (**a**–**c**) are examples of newly predicted target proteins of drugs (see Supplementary Fig. S9). Blue circles denote drugs, red rectangles denote target proteins, green diamonds indicate diseases, and gray edges and red lines denote known interactions and newly predicted interactions, respectively.

Figure 6 shows a small part of the drug–protein–disease network consisting of newly predicted drug–protein interactions and therapeutic indications. We examined the drug–protein–disease relationships that were predicted only by our proposed method (that were not predicted by the previous methods). We discuss several examples of the prediction results for highlighted drugs in Fig. 6a, b and c.

Figure 6a shows examples of target proteins predicted for loratadine (D00364), which is a marketed anti-histamine (see Supplementary Fig. S9a for more details). Loratadine was predicted to interact with interleukin 3 (IL3; hsa:3562) based on its transcriptional similarity (score = 0.838) with amlexanox (D01828), where both gene expression signatures were obtained from the A375 cell line derived from a melanoma. These two drugs are different in terms of both efficacy and chemical structure. Although both drugs are anti-allergic drugs, loratadine is an anti-histamine and amlexanox is a chemical mediator release inhibitor. The chemical structure similarity score of the two drugs is only 0.170. Nevertheless, these two drugs have some downstream effects in common: loratadine acts as a histamine H1-receptor antagonist and inhibits the secretion of IL3^28^, whereas amlexanox inhibits the release of mediators such as histamine^29^, which plays a central role in allergic reactions^30^, and also inhibits IL3, which stimulates the release of histamine^31^. Therefore, the predicted interaction is quite likely to occur. In fact, we were able to confirm the validity of the interaction in the literature^28^. Our pathway enrichment analysis identified the Fc*ε*RI signaling pathway (hsa04664), which is known to be a major pathway in an allergic reaction, as a pathway in which the genes were inactivated. This result suggests that differently categorized anti-allergic drugs have similar gene expression patterns and the same allergy-associated pathways.

Figure 6b shows examples of target proteins predicted for rosiglitazone (D08491), which is a marketed anti-diabetic (see Supplementary Fig. S9b for more details). Rosiglitazone was transcriptionally similar to thalidomide (D00754), which is used to treat multiple myeloma. These two drugs induced similar gene expression patterns (score = 0.732), although they differ in chemical structure (score = 0.306). The transcriptional similarity score was calculated based on gene expression signatures in the promonocytic leukemia NOMO1 cell line. The NOMO1 cell line was available only in LINCS, so this prediction was not possible using CMap. Using independent resources, we were able to confirm the validity of the interaction between rosiglitazone and NFKB1^32^ and also the inhibition of NFKB1 activity by thalidomide^33^, which leads to the prevention of diabetic retinopathy in diabetic rats^34^. These results suggest that rosiglitazone may have anti-inflammatory and anti-cancer effects, in addition to its known ability to decrease blood sugar levels.

Figure 6c shows an example of the target proteins predicted for phenothiazine (D02601), an anthelmintic and an anti-psychotic drug (see Supplementary Fig. S9c for more details). Phenothiazine was predicted to interact with the androgen receptor (AR; hsa:367) based on its transcriptional similarity with enzalutamide (D10218), which is marketed for the treatment of prostate cancer and is known to interact with AR. Although the two drugs are chemically dissimilar (score = 0.077), they induced similar gene expression signatures (score = 0.905) in the HCT116 cell line. In fact, enzalutamide antagonizes AR to inhibit the androgen-induced proliferation of metastatic, castration-resistant prostate cancer cells, and enzalutamide induces apoptosis for prostate cancer cells in which AR is highly expressed^35^. As shown in Supplementary Fig. S9d, the apoptosis pathway was detected for both enzalutamide and phenothiazine. These results suggest that phenothiazine might work for prostate cancer in a similar manner to that of enzalutamide.

### Experimental validation by an *in vitro* cellular assay

We performed an *in vitro* cellular assay to experimentally test the prediction that phenothiazine inhibits AR. In this set of experiments, HEK293 cells were transformed with the plasmids used in the GAL4 assay system of Phenex Pharmaceuticals AG. In these cells, interaction with the androgen receptor is coupled to the production of *Photinus pyralis* luciferase. When run in agonist mode, higher luminescence is observed at higher agonist concentrations, whereas in the antagonist mode, lower luminescence is observed at higher antagonist concentrations.

**Figure 7:**
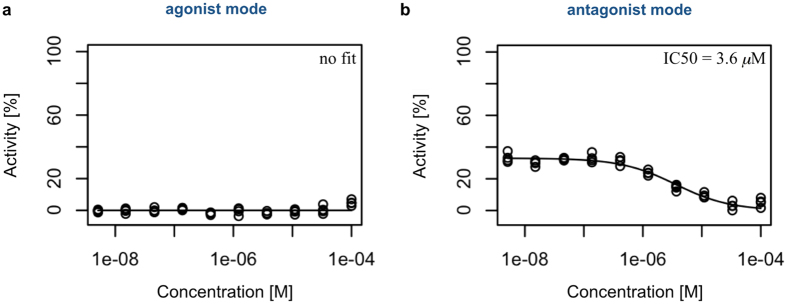
Dose response curves of phenothiazine in the AR-binding assay run in (**a**) agonist and (**b**) antagonist modes. The horizontal axis represents the concentrations of phenothiazine on a logarithmic scale, and the vertical axis represents the percentages of phenothiazine activity. The open circles represent the data points from triplicate experiments.

Figure 7a and b show the dose response curves of phenothiazine from the assay run in agonist and antagonist modes, respectively. It was observed that phenothiazine had a strong antagonistic effect, yielding an IC50 of 3.6 *μ*M. The percentage of activity decreased from approximately 35% to 5%. No strong effects were observed for the other drugs (see Supplementary Fig. S10). These experimental results validate the prediction that phenothiazine inhibits signal transduction through the androgen receptor and may, therefore, be useful for the treatment of prostate cancer.

## Discussion

In this paper, we proposed a novel computational approach for elucidating the modes of action of bioactive compounds from large-scale chemically-induced transcriptome data acquired from LINCS. This approach makes it possible to predict active pathways, target proteins, and therapeutic indications in a seamless manner. The originality of the approach lies in a similarity search that is based on cell-specific transcriptional similarity, in its ability to make simultaneous predictions of thousands of candidate target proteins and candidate disease indications, and in its lack of dependence upon compound chemical structures. We demonstrated the usefulness of the proposed approach in terms of prediction coverage, interpretability, and large-scale applicability. The proposed approach is expected to be useful for drug discovery and drug repositioning.

Drug discovery is facilitated by a deep understanding of the modes of action of candidate drug compounds. Phenotype-based high-throughput screening (PHTS) is useful for finding candidate drug compounds that have a desired phenotype. However, the underlying molecular mechanisms employed by hit compounds identified by PHTS remain unknown, and considerable effort is usually required to identify the target proteins associated with the phenotype. Obtaining information on the mechanisms of drug action (e.g., the primary target, off-targets, and pathway activities) can help to infer potential therapeutic effects or side effects. Cellular transcriptomic responses give us mechanistic insights into the modes of action of bioactive compounds, so the proposed approach will help researchers to efficiently predict the phenotype-associated target proteins of drug candidate compounds. In this study, we focused on the use of compound-induced gene expression data for mechanistic insights. Most chemoinformatics methods for target prediction are based on compound chemical structures (e.g., QSAR). However, it is impossible to obtain the information on the mechanism. It is also difficult to predict compound–target interactions that could not be expected from compound chemical structures. If compounds in the learning set were biased, predictable target proteins would also be biased.

During the pathway enrichment analysis, we focused on genes that were upregulated or downregulated genes by chemical perturbation, and identified pathways in which upregulated and downregulated genes were significantly enriched. Although the target proteins of numerous bioactive compounds (including approved drugs) have not been identified yet, the proposed method can suggest pathway activities triggered by the compound target, which might yield clues for identifying the target proteins related to the observed phenotype. However, the performance of the method depends heavily on the quality and coverage of biological pathways and the threshold values for determining upregulated and downregulated genes. In this study, we extracted the top and bottom 5% of genes as upregulated and downregulated genes, respectively, but the choice of an appropriate threshold value is an important area for future work.

We addressed the problem of using various cell lines in the analysis of chemically-induced transcriptome data. As shown in Fig. 1, each cell line has specific gene expression patterns; thus, there is a need to distinguish gene expression signatures between various cell lines. It has been difficult to obtain gene expression profiles from the same cell line between different compounds, but LINCS resource covers a large number of cell lines (68 cell lines in this study). This means that LINCS is a valuable and comprehensive transcriptome resource. In fact, the prediction results were more reliable when analyzing gene expression signatures obtained from the same cell line, suggesting the importance of appropriately selecting cell lines. This knowledge will contribute to the policy for consolidating more comprehensive transcriptome databases in the future.

It is shown that LINCS has superiority over CMap in terms of data coverage, but there are weak correlation of gene expression signatures between CMap and LINCS. Supplementary Fig. S11 shows histograms displaying the similarity of transcriptional responses obtained for the same compounds and the same cell lines (i.e., MCF7 and PC3) using data from CMap and LINCS. The similarity scores of some compounds were relatively high, but most similarity scores were distributed around 0.0. This observation might stem from differences in the experimental technologies used to measure transcript levels (i.e., microarray for CMap and flow cytometry for LINCS). A limitation of the method in this study is that it cannot distinguish whether compound–target interactions result in activation or inhibition. The integrative use of genetically perturbed gene expression data (e.g., gene knockdown, gene overexpression) would be a promising approach to improve the reliability of the methods.

## Methods

### Chemically perturbed transcriptome data from LINCS

Gene expression profiles from the LINCS project were obtained from the Broad Institute’s website (http://www.lincscloud.org). This project is based on 77 human cell lines and various cellular perturbations, including compound treatment, gene knockdown, gene overexpression, and several more interventions. In this study we used the data from the 68 cell lines used in compound treatment experiments. Gene expression levels were measured using flow cytometry^36^, and test samples were prepared using 384-well plates. LINCS provides 978 landmark genes called “L1000 genes.” Out of 978 landmark genes in this study, 614 genes are essential genes according to the DEG database^37^. The expression levels of these landmark genes were experimentally measured^21^. The expression levels of the remaining genes (approximately 21,000 genes) were estimated using a computational model based on the Gene Expression Omnibus^38^. The gene expression values for the landmark genes were used in this study.

Gene expression levels were measured at 6 h, 6.4 h, 24 h, 24.4 h, and 48 h after compound treatment. Each gene expression profile was filed using its “distil_id.” The total number of profiles was 1,328,098. We first selected 663,594 compound treatment profiles (denoted “trt_cp” in the information) and 28,557 control profiles (denoted “ctl_vehicle”). We determined the correspondence between the compound treatment profiles and the control profiles by comparing their “distil_ids.” We excluded gene expression profiles that lacked corresponding control profiles. The name of each compound was converted into its corresponding InChIKey (http://www.iupac.org/home/publications/e-resources/inchi.html) via the perturbation ID using the information provided in LINCS, which yielded the gene expression profiles of 71 cell lines treated with 20,122 bioactive compounds. In this study, we used the gene expression profiles, measured at 6 (6.4) h, of 68 cell lines treated with 16,268 bioactive compounds.

### Chemically perturbed transcriptome data from CMap

Gene expression profiles in CMap (build 02) were obtained from the Broad Institute’s website (http://www.broadinstitute.org/cmap). CMap contains 7,056 profiles based on five cell lines treated with 1,309 compounds. Gene expression levels were measured at 6 h after compound treatment. The gene expression profiles of 22,277 genes were experimentally measured using microarray technology. We selected some profiles measured using the HT_HG-U133A platform and the human cell lines MCF7 (breast adenocarcinoma), PC3 (prostate cancer), and HL60 (promyelocytic leukemia). The name of each compound was converted into its corresponding InChIKey via ChemBank^39^, which yielded the gene expression signatures of 1,133 chemical compounds. We also prepared gene expression profiles consisting of the 978 landmark genes (L1000 genes) from LINCS^21^ to enable comparisons between CMap and LINCS.

### Chemically perturbed transcriptome data from TG-GATEs

Gene expression profiles in TG-GATEs were obtained from the Toxicogenomics Project (TGP) of Japan (http://toxico.nibio.go.jp/english/). TGP is based on only 170 compounds and one primary hepatocyte *in vitro* (rat or human). The name of each compound was converted into its corresponding InChIKey, which yielded the gene expression signatures of 157 compounds.

### Construction of gene expression signatures

In the present study, we constructed chemically-induced gene expression profiles, which we call “gene expression signatures” or “signatures” for short. A gene expression signature is a high-dimensional feature vector in which each element is defined as the logarithmic ratio of the gene expression value measured after compound treatment to that measured in the corresponding control condition. In LINCS, gene expression profiles denoted by “Level 3” and “Level 4” are available. Level 3 data consist of profiles generated using invariant-set scaling and quantile normalization. Level 4 data consist of profiles generated using robust z-scores relative to population controls generated with the average over all other wells on the 384-well plate.

We applied two types of normalization procedures: “biological control” and “mean centering.” During “biological control” normalization, a gene expression signature was constructed based on the logarithmic ratio of the compound treatment profile to the biological control profile^8^. Then, it was centered to have a mean of zero and scaled to have standard deviation of one. For the LINCS data, we used Level 3 data. For the CMap data, we applied the Robust Multiarray Average (RMA)^40^ method to the original data in Affymetrix CEL file format.

During “mean centering” normalization, a control profile was prepared by averaging compound treatment profiles measured under the same biological control conditions, which is referred to as the averaged control profile. A gene expression signature is constructed based on the logarithmic ratio of the compound treatment profile to the averaged control profile. Then, it was centered to have mean zero and scaled to have a standard deviation of one. The mean centering normalization procedure is known to reduce batch effects in CMap^17^. In the case of LINCS, we used Level 4 data, because a similar approach was applied to Level 4 data generation.

We represented the gene expression signature of each compound with a feature vector as 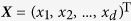, where *d* is the number of features and the number of features is identical to the number of genes. For each cell line, the same compound has multiple signatures based on various concentrations and different time points. In this study, multiple signatures were integrated into a single signature by averaging the signatures, and 6.4 h and 24.4 h were regarded as 6 h and 24 h, respectively.

### Top to bottom gene selection from signatures

Small variations in gene expression values may be noise. The use of the expression ratios only for top-ranked and bottom-ranked genes has been proposed in previous studies^12^,^13^,^17^,^18^,^19^,^20^. The expression ratios of the remaining genes were assigned values of zero. We tested using top 50 and bottom 50 ranked genes, the top 100 and bottom 100 ranked genes, and all genes, which are referred to as “top50”, “top100”, and “all”, respectively.

### Chemical structures

The chemical structures of the compounds were obtained from ChEMBL^41^, and were represented by their KEGG Chemical Function and Substructures (KCF-S) descriptors^42^. Each compound was coded by a high-dimensional feature vector, in which each element indicates the frequency of a feature defined by KCF-S (i.e., chemical substructures). The number of features was 475,692. We computed chemical structure similarity scores of compounds using the generalized Jaccard correlation coefficient. Note that the original Jaccard coefficient (Tanimoto coefficient) can only treat binary vectors (fingerprints).

### Chemical–protein interactome

Compound–protein interaction data were acquired from seven databases: ChEMBL^41^, MATADOR^43^, DrugBank^44^, the Psychoactive Drug Screening Program Ki^45^, KEGG^24^, the Binding DB^46^, and the Therapeutic Target Database^47^. For the ChEMBL data, we selected only compound–protein interaction pairs that were clearly denoted as active interactions or with binding affinities of <30 *μ*M (e.g., IC50), which yielded 1,287,404 compound–protein interactions involving 519,061 compounds and 3,735 proteins. This dataset is referred to as “chemical–protein interactome data”. Out of 3,735 genes of target candidate proteins in this study (predictable target proteins), 1,813 genes are essential genes according to the DEG database^37^.

To evaluate the performance of compound target prediction, we prepared two benchmark datasets: common data and merged data. Common data consisted of compound–protein interactions involving compounds common to CMap and LINCS. This dataset is comprised of 391 compounds, 1,377 target proteins, and 7,686 interacting pairs. Merged data consisted of compound–protein interactions involving chemical compounds in CMap or LINCS. This dataset is comprised of 3,767 compounds, 2,419 target proteins, and 38,129 interacting pairs.

### Therapeutic indications

Therapeutic indications can be regarded as drug–disease associations. Drug–disease association data were obtained from medical books^48^ and KEGG DISEASE^24^. Diseases defined in ICD-10 were used in this study. In total, 5,606 drug–disease associations involving 2,266 drugs and 461 diseases were obtained.

### Pathway enrichment analyses

Pathway enrichment analyses of upregulated and downregulated genes were performed following the procedures used in previous studies for different biological problems^49^,^50^. We used the 163 biological pathways in the following categories in KEGG: Metabolism (except for Global and overview maps), Environmental Information Processing (except for Membrane transport and Signaling molecules and interaction), Cellular Processes (except for Transport and catabolism), and Organismal Systems. To perform the analysis, we used the genes ranked in the top 5% and the bottom 5% of genes. We analyzed not only interactome compounds (of known targets) but also other compounds (of unknown targets) for which gene expression data are available.

Let *G*_compound_ denote a set of upregulated or downregulated genes in a signature induced by a compound, and let *G*_pathway_ denote a set of genes in a pathway map. Further, let *r* = 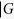
_compound_|, *k* = |*G*_pathway_|, *z* = |*G*_compound_ ∩ *G*_pathway_| and *l* the total number of genes in the entire dataset (*l* = 978). We assumed that *z* follows a hypergeometric distribution. The probability of observing an intersection of size *z* between *G*_pathway_ and *G*_compound_ is computed as follows:


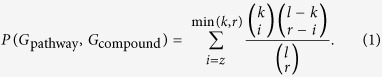


The resulting *P*-values were corrected using the false discovery rate (FDR)^51^.

### Target protein prediction

We predicted which proteins are potential targets of query compounds using a similarity search based on gene expression signatures and large-scale chemical–protein interactome data. Compounds and proteins included in the chemical–protein interactome data are referred to as interactome compounds and interactome proteins, respectively. The details of the similarity search are described in the next section.

A query compound is represented by the feature vector 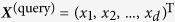 and each interactome compound is represented by the feature vector 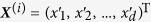, where 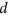 is the number of genes. Each interactome compound is represented by a target-protein interaction profile 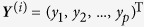, where 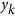 indicates the presence or absence of an interaction with the *k*th protein by 1 or 0 (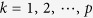), and *p* is the number of interactome proteins.

First, we compute pairwise similarity scores for all pairs between a given query compound and all of the interactome compounds in our chemical–protein interactome data. Second, from the interactome compounds known to interact with the *k*th protein (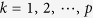), we select an interactome compound with the highest similarity to the query compound and use the corresponding similarity score as a prediction score to assess the possibility that the query compound interacts with the *k*th protein. Third, we repeat this procedure for all *p* interactome proteins and assign the prediction scores to compound–protein pairs (pairs between the query compound and all interactome proteins). Finally, high scoring compound–protein pairs are predicted as candidates for interaction pairs.

### Cell-based similarity search

The transcriptional similarity between two gene expression signatures ***X*** and 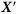 is calculated using the Pearson correlation coefficient as follows:


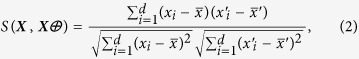


where 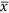 and 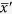 represent the means of the gene expression values in vectors ***X*** and 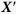, respectively.

Because gene expression data differ from cell line to cell line, we propose a cell-based similarity search based upon the following two strategies. In the first strategy, called same cell line-matching, we calculated the transcriptional similarity score between a query compound 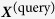 and an interactome compound 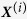 based on the same cell lines as follows:


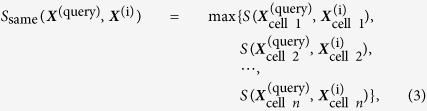


where 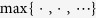 is an operation to take the maximum value and *n* is the number of cell lines. In practice, gene expression data are not always available from the same cell line, so we also used the second strategy, called different cell line-matching. In the different cell line-matching strategy, we calculated the transcriptional similarity score between a query compound 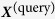 and an interactome compound 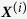 based on different cell lines as follows:


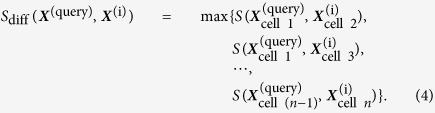


We also tested the integration of the above two matching strategies, taking the maximal value between 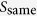 and 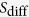, which we call “all cell line-matching strategy” (see Supplementary Method).

### Therapeutic indication prediction

We predicted new therapeutic indications for query compounds based on the results of the compound–target interaction predictions. In advance, we prepared large-scale drug–disease association data. If the query compound has high transcriptional similarity with interactome compounds of known indications and shares the same target proteins with the interactome compounds, the query compound is predicted to have the same therapeutic indications as the most similar compound. The prediction scores for compound–disease associations are assigned the same prediction scores as the associated compound–protein interactions. We used the prediction scores as weights on newly predicted links in the drug-target-disease network. Highly weighted links correspond to strong links (high-confidence prediction).

### *In vitro* cellular assay

An *in vitro* cellular GAL4 gene reporter assay was performed by Phenex Pharmaceuticals AG. Phenothiazine (Sigma-Aldrich, #46624) was tested against the human androgen receptor (AR). The assay was performed in two modes, agonist and antagonist. In the agonist mode, higher levels of luminescence were observed for higher concentrations of agonists, whereas in the antagonist mode, lower levels of luminescence were observed for higher concentrations of antagonists. The compounds were tested at 10 concentrations in triplicates. The solutions of the compounds were freeze/thawed three times and stored at −20 °C in dimethyl sulfoxide (DMSO).

HEK293 cells (DSMZ ACC 305) were transformed with the plasmids used in the Phenex GAL4 assay system. The plasmids were derivatives of the Stratagene M2H reporter plasmid; the reporter plasmids were a modified pFR-Luc, containing a synthetic promoter with two tandem repeats of the yeast GAL4-binding sites that control the expression of the *Photinus pyralis* luciferase gene, and pCMV-BD. For the AR-binding assay, a plasmid constitutively expressing a PGC1*α* fragment was also used to obtain reasonable assay quality. A second reporter (pRL-CMV), with *Renilla reniformis* luciferase driven by a constitutive promoter, was also used to improve the accuracy of the experiments.

The *Photinus* luciferase activity value was divided by the *Renilla* luciferase activity value and multiplied by 1,000 (the resulting value is denoted as “RENnorm”). RENnorm was calculated for each well and used for the calculation of percent activity values as follows:


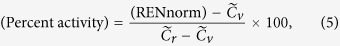


where 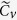 and 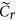 represent the median values for DMSO vehicle (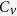; negative control) and a reference compound (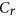; positive control), respectively, calculated per assay plate. The activity in the negative control wells (DMSO vehicle only) equals to 0%, and that in the positive control wells (saturating concentration of a reference compound) equals to 100%. The reference compounds were dihydrotestosterone (C03917) and bicalutamide (D00961) for the agonist and antagonist modes, respectively.

The R package drc^52^ was used to calculate the dose response curves and EC50/IC50 values using a three-parameter logistic function. To draw a dose response curve for triflupromazine hydrochloride, it was necessary to exclude the data point generated at the highest concentration (100 *μ*M).

## Additional Information

**How to cite this article:** Iwata, M. *et al*. Elucidating the modes of action for bioactive compounds in a cell-specific manner by large-scale chemically-induced transcriptomics. *Sci. Rep.* **7**, 40164; doi: 10.1038/srep40164 (2017).

**Publisher's note:** Springer Nature remains neutral with regard to jurisdictional claims in published maps and institutional affiliations.

## Supplementary Material

Supplementary Information

## Figures and Tables

**Table 1:** Evaluation of target protein prediction using common data (a) and merged data (b).

a. common data							
**similarity search strategy**	CMap - Biological control					LINCS - Level 3	
	signature : all genes		signature : L1000 genes		signature : L1000 genes		
	AUC	AUPR	AUC	AUPR	AUC	AUPR	
same cell line-matching	0.8288	0.1213	0.8310	0.1279	0.8203	0.1145	
different cell line-matching	0.8178	0.1027	0.8283	0.1161	0.8099	0.0922	
**similarity search strategy**	CMap - Mean centering				LINCS - Level 4		
	signature : all genes		signature : L1000 genes		signature : L1000 genes		
	AUC	AUPR	AUC	AUPR	AUC		AUPR
same cell line-matching	0.8261	0.1228	0.8257	0.1248	0.8145		0.1115
different cell line-matching	0.8176	0.1036	0.8224	0.1179	0.8077		0.0992
**b. merged data**							
**similarity search strategy**	CMap - Biological control				LINCS - Level 3		
	signature : all genes		signature : L1000 genes		signature : L1000 genes		
	AUC	AUPR	AUC	AUPR	AUC		AUPR
same cell line-matching	0.5648	0.0232	0.5650	0.0241	0.8608		0.0985
different cell line-matching	0.5640	0.0206	0.5649	0.0228	0.8195		0.0505
**similarity search strategy**	CMap - Mean centering				LINCS - Level 4		
	signature : all genes		signature : L1000 genes		signature : L1000 genes		
	AUC	AUPR	AUC	AUPR	AUC		AUPR
same cell line-matching	0.5643	0.0235	0.5643	0.0231	0.8517		0.0819
different cell line-matching	0.5641	0.0206	0.5645	0.0215	0.8245		0.0537

## References

[b1] Whitebread S., Hamon J., Bojanic D. & Urban L. Keynote review: *in vitro* safety pharmacology profiling: an essential tool for successful drug development. Drug Discov. Today 10, 1421–1433 (2005). 16243262 10.1016/S1359-6446(05)03632-9

[b2] Blagg J. Structure-activity relationships for *in vitro* and *in vivo* toxicity. Annu. Rep. Med. Chem. 41, 353–368 (2006).

[b3] Lin S.-F., Xiao K. T., Huang Y. T., Chiu C. C. & Soo V. W. Analysis of adverse drug reactions using drug and drug target interactions and graph-based methods. Artif. Intell. Med. 48, 161–166 (2010). 19962282 10.1016/j.artmed.2009.11.002

[b4] Ashburn T. T. & Thor K. B. Drug repositioning: identifying and developing new uses for existing drugs. Nat. Rev. Drug Discov. 3, 673–683 (2004). 15286734 10.1038/nrd1468

[b5] Chong C. R. & Sullivan D. J. New uses for old drugs. Nature 448, 645–646 (2007). 17687303 10.1038/448645a

[b6] Novac N. Challenges and opportunities of drug repositioning. Trends Pharmacol. Sci. 34, 267–272 (2013). 23582281 10.1016/j.tips.2013.03.004

[b7] Igarashi Y. . Open TG-GATEs: a large-scale toxicogenomics database. Nucleic Acids Res. 43, D921–D927 (2015). 25313160 10.1093/nar/gku955 PMC4384023

[b8] Lamb J. . The Connectivity Map: using gene-expression signatures to connect small molecules, genes, and disease. Science 313, 1929–1935 (2006). 17008526 10.1126/science.1132939

[b9] Dudley J. T. . Computational repositioning of the anticonvulsant topiramate for inflammatory bowel disease. Sci. Transl. Med. 3, 96ra76 (2011). 10.1126/scitranslmed.3002648 PMC3479650 21849664

[b10] Kosaka T. . Identification of drug candidate against prostate cancer from the aspect of somatic cell reprogramming. Cancer Sci. 104, 1017–1026 (2013). 23600803 10.1111/cas.12183 PMC7657195

[b11] van Noort V. . Novel drug candidates for the treatment of metastatic colorectal cancer through global inverse gene-expression profiling. Cancer Res. 74, 5690–5699 (2014). 25038229 10.1158/0008-5472.CAN-13-3540

[b12] Iorio F., Tagliaferri R. & di Bernardo D. Identifying network of drug mode of action by gene expression profiling. J. Comput. Biol. 16, 241–251 (2009). 19183001 10.1089/cmb.2008.10TT

[b13] Iorio F. . Discovery of drug mode of action and drug repositioning from transcriptional responses. Proc. Natl. Acad. Sci. USA 107, 14621–14626 (2010). 20679242 10.1073/pnas.1000138107 PMC2930479

[b14] Hizukuri Y., Sawada R. & Yamanishi Y. Predicting target proteins for drug candidate compounds based on drug-induced gene expression data in a chemical structure-independent manner. BMC Med. Genomics 8, 82 (2015). 26684652 10.1186/s12920-015-0158-1 PMC4683716

[b15] Iskar M. . Characterization of drug-induced transcriptional modules: towards drug repositioning and functional understanding. Mol. Syst. Biol. 9, 662 (2013). 23632384 10.1038/msb.2013.20 PMC3658274

[b16] Parkkinen J. A. & Kaski S. Probabilistic drug connectivity mapping. BMC Bioinformatics 15, 113 (2014). 24742351 10.1186/1471-2105-15-113 PMC4011783

[b17] Iskar M. . Drug-induced regulation of target expression. PLoS Comput. Biol. 6, e1000925 (2010). 20838579 10.1371/journal.pcbi.1000925 PMC2936514

[b18] Wang K. . Prediction of drug-target interactions for drug repositioning only based on genomic expression similarity. PLoS Comput. Biol. 9, e1003315 (2013). 24244130 10.1371/journal.pcbi.1003315 PMC3820513

[b19] Cheng J. . Evaluation of analytical methods for connectivity map data. Pac. Symp. Biocomput., 5–16 (2013). 23424107

[b20] Cheng J., Yang L., Kumar V. & Agarwal P. Systematic evaluation of connectivity map for disease indications. Genome Med. 6, 540 (2014). 25606058 10.1186/s13073-014-0095-1 PMC4278345

[b21] Duan Q. . LINCS Canvas Browser: interactive web app to query, browse and interrogate LINCS L1000 gene expression signatures. Nucleic Acids Res. 42, W449–W460 (2014). 24906883 10.1093/nar/gku476 PMC4086130

[b22] Liu C. . Compound signature detection on LINCS L1000 big data. Mol. Biosyst. 11, 714–722 (2015). 25609570 10.1039/c4mb00677a PMC4333019

[b23] Chen B. . Relating chemical structure to cellular response: an integrative analysis of gene expression, bioactivity, and structural data across 11,000 compounds. CPT Pharmacometrics Syst. Pharmacol. 4, 576–584 (2015). 26535158 10.1002/psp4.12009 PMC4625862

[b24] Kanehisa M., Goto S., Furumichi M., Tanabe M. & Hirakawa M. KEGG for representation and analysis of molecular networks involving diseases and drugs. Nucleic Acids Res. 38, D355–D360 (2010). 19880382 10.1093/nar/gkp896 PMC2808910

[b25] Liu J.-D. . Molecular mechanisms of G0/G1 cell-cycle arrest and apoptosis induced by terfenadine in human cancer cells. Mol. Carcinog. 37, 39–50 (2003). 12720299 10.1002/mc.10118

[b26] Fabregat A. . The Reactome pathway Knowledgebase. Nucleic Acids Res. 44, D481–D487 (2016). 26656494 10.1093/nar/gkv1351 PMC4702931

[b27] World Health Organization The ICD-10 Classification of Mental and Behavioral Disorders: Clinical Descriptions and Diagnostic Guidelines. Geneva: World Health Organization (1992).

[b28] Lippert U., Möller A., Welker P., Artuc M. & Henz B. M. Inhibition of cytokine secretion from human leukemic mast cells and basophils by H1-and H2-receptor antagonists. Exp. Dermatol. 9, 118–124 (2000). 10772385 10.1034/j.1600-0625.2000.009002118.x

[b29] Bell J. Amlexanox for the treatment of recurrent aphthous ulcers. Clin. Drug Investig. 25, 555–566 (2005). 10.2165/00044011-200525090-00001 17532700

[b30] Bachert C. Histamine - a major role in allergy? Clin. Exp. Allergy 28, 15–19 (1998). 10.1046/j.1365-2222.1998.0280s6015.x 9988429

[b31] Ferrer M., Luquin E. & Kaplan A. P. IL3 effect on basophils histamine release upon stimulation with chronic urticaria sera. Allergy 58, 802–807 (2003). 12859562 10.1034/j.1398-9995.2003.00195.x

[b32] Zhou X. & You S. Rosiglitazone inhibits hepatic insulin resistance induced by chronic pancreatitis and IKK-*β*/NF-*κ*B expression in liver. Pancreas 43, 1291–1298 (2014). 25036911 10.1097/MPA.0000000000000173

[b33] Yasui K., Kobayashi N., Yamazaki T. & Agematsu K. Thalidomide as an immunotherapeutic agent: the effects on neutrophil-mediated inflammation. Curr. Pharm. Des. 11, 395–401 (2005). 15723633 10.2174/1381612053382179

[b34] Bosco A. A., Lerario A. C., Santos R. F. & Wajchenberg B. L. Effect of thalidomide and rosiglitazone on the prevention of diabetic retinopathy in streptozotocin-induced diabetic rats. Diabetologia 46, 1669–1675 (2003). 14598030 10.1007/s00125-003-1234-1

[b35] Tran C. . Development of a second-generation antiandrogen for treatment of advanced prostate cancer. Science 324, 787–790 (2009). 19359544 10.1126/science.1168175 PMC2981508

[b36] Peck D. . A method for high-throughput gene expression signature analysis. Genome Biol. 7, R61 (2006). 16859521 10.1186/gb-2006-7-7-r61 PMC1779561

[b37] Zhang R., Ou H.-Y. & Zhang C.-T. DEG: a database of essential genes Nucleic Acids Res. 32, D271–D272 (2004). 14681410 10.1093/nar/gkh024 PMC308758

[b38] Barrett T. . NCBI GEO: mining tens of millions of expression profiles-database and tools update. Nucleic Acids Res. 35, D760–D765 (2007). 17099226 10.1093/nar/gkl887 PMC1669752

[b39] Seiler K. P. . ChemBank: a small-molecule screening and cheminformatics resource database. Nucleic Acids Res. 36, D351–D359 (2008). 17947324 10.1093/nar/gkm843 PMC2238881

[b40] Irizarry R. A. . Summaries of Affymetrix GeneChip probe level data. Nucleic Acids Res. 31, e15 (2003). 12582260 10.1093/nar/gng015 PMC150247

[b41] Gaulton A. . ChEMBL: a large-scale bioactivity database for drug discovery. Nucleic Acids Res. 40, D1100–D1107 (2012). 21948594 10.1093/nar/gkr777 PMC3245175

[b42] Kotera M. . KCF-S: KEGG Chemical Function and Substructure for improved interpretability and prediction in chemical bioinformatics. BMC Syst. Biol. 7, S2 (2013). 10.1186/1752-0509-7-S6-S2 PMC4029371 24564846

[b43] Günther S. . SuperTarget and Matador: resources for exploring drug-target relationships. Nucleic Acids Res. 36, D919–D922 (2008). 17942422 10.1093/nar/gkm862 PMC2238858

[b44] Knox C. . DrugBank 3.0: a comprehensive resource for ‘omics’ research on drugs. Nucleic Acids Res. 39, D1035–D1041 (2011). 21059682 10.1093/nar/gkq1126 PMC3013709

[b45] Roth B. L., Lopez E., Patel S. & Kroeze W. K. The multiplicity of serotonin receptors: uselessly diverse molecules or an embarrassment of riches? Neuroscientist 6, 252–262 (2000).

[b46] Liu T., Lin Y., Wen X., Jorissen R. N. & Gilson M. K. BindingDB: a web-accessible database of experimentally determined protein-ligand binding affinities. Nucleic Acids Res. 35, D198–D201 (2007). 17145705 10.1093/nar/gkl999 PMC1751547

[b47] Zhu F. . Therapeutic target database update 2012: a resource for facilitating target-oriented drug discovery. Nucleic Acids Res. 40, D1128–D1136 (2012). 21948793 10.1093/nar/gkr797 PMC3245130

[b48] Papadakis M. A., McPhee S. J. & Rabow M. W. *Current Medical Diagnosis and Treatment 2014.* McGraw Hill Medical (2014).

[b49] Mizutani S., Pauwels E., Stoven V., Goto S. & Yamanishi Y. Relating drug-protein interaction network with drug side effects. Bioinformatics 28, i522–i528 (2012). 22962476 10.1093/bioinformatics/bts383 PMC3436810

[b50] Hung J.-H. Gene set/pathway enrichment analysis. Methods Mol. Biol. 939, 201–213 (2013). 23192548 10.1007/978-1-62703-107-3_13

[b51] Benjamini Y. & Hochberg Y. Controlling the false discovery rate: a practical and powerful approach to multiple testing. J. R. Statist. Soc. B 57, 289–300 (1995).

[b52] Ritz C. & Streibig J. C. Bioassay analysis using R. J. Stat. Softw. 12, 1–22 (2005).

