# Supplementary material for: Elucidating the modes of action for bioactive compounds in a cell-specific manner by large-scale chemically-induced transcriptomics
Source: Sci Rep. 2017 Jan 10;7:40164. doi: 10.1038/srep40164 (PMC5223214; doi:10.1038/srep40164)
Supplement: Supplementary Information [file srep40164-s1.pdf]

Supplementary information for

Elucidating the modes of action for bioactive compounds in a cell-specific manner by large-scale chemically-induced transcriptomics

Michio Iwata[1], Ryusuke Sawada[1], Hiroaki Iwata[1], Masaaki Kotera[2], Yoshihiro Yamanishi[1,3,4,*]

1. Division of System Cohort, Medical Institute of Bioregulation, Kyushu University, 3-1-1 Maidashi, Higashi-ku, Fukuoka, Fukuoka 812-8582, Japan. 2. School of Life Science and Technology, Tokyo Institute of Technology, 2-12-1 Ookayama, Meguro-ku, Tokyo, 152-8550, Japan. 3. Institute for Advanced Study, Kyushu University, 3-1-1 Maidashi, Higashi-ku, Fukuoka, Fukuoka 812-8582, Japan. 4. PRESTO, Japan Science and Technology Agency, Kawaguchi, Saitama 332-0012, Japan.
*corresponding author: Yoshihiro Yamanishi (yamanishi@bioreg.kyushu-u.ac.jp)

Supplementary Method

All cell line-matching strategy

In the all cell line-matching strategy, we calculated the transcriptional similarity score between a query compound $\boldsymbol{X}^{(\mathrm{query})}$ and an interactome compound $\boldsymbol{X}^{(\mathrm{i})}$ based on the integration of the same cell lines and different cell lines as follows:

$$S_{\mathrm{all}}(\boldsymbol{X}^{(\mathrm{query})}, \boldsymbol{X}^{(\mathrm{i})}) = \max\{S_{\mathrm{same}}(\boldsymbol{X}^{(\mathrm{query})}, \boldsymbol{X}^{(\mathrm{i})}), S_{\mathrm{diff}}(\boldsymbol{X}^{(\mathrm{query})}, \boldsymbol{X}^{(\mathrm{i})})\}. \quad \text{(S.1)}$$

Supplementary Figures Legends

Supplementary Figure S1 Venn diagram of cell lines (left) and chemical compounds (right) present in TG-GATEs, CMap, and LINCS; the three databases containing chemically-induced gene expression profiles.

Supplementary Figure S2 Landmark genes of the cell cycle that are partially downregulated by drug perturbations. Information concerning drugs and cell lines are shown in the first three columns. Drugs are categorized according to the ATC code. Names of drugs and genes are given in alphabetical order. Downregulated genes are colored green. Code J: anti-infectives for systemic use; code L: anti-neoplastic and immunomodulating agents; code N: nervous system; code R: respiratory system; and code S: sensory organs.

Supplementary Figure S3 Distribution of drug classifications according to the biological pathways that they activate (top) and inactivate (bottom). The fraction of drugs in a particular classification that affect each pathway is represented by the intensity of color in the appropriate box. The intensity of color indicates the relative frequency (the compound frequency was divided by the number of compounds in each Anatomical Therapeutic Chemical classification system (ATC code)). The boxes are arranged according to the first level of the ATC code. Drug are assigned the following ATC codes: code A: alimentary tract and metabolism; code B: blood and blood-forming organs; code C: cardiovascular system; code D: dermatologicals; code G: genitourinary system and sex hormones; code H: systemic hormonal preparations, excluding sex hormones and insulins; code J: anti-infectives for systemic use; code L: anti-neoplastic and immunomodulating agents; code M: musculo-skeletal system; code N: nervous system; code P: anti-parasitic products, insecticides and repellents; code R: respiratory system; code S: sensory organs; and code V: various.

Supplementary Figure S4 Distribution of the identified pathways in REACTOME. (a) the histogram of detected pathways by the result of analyzing all compounds, where the horizontal axis indicates the list of biological pathways and the vertical axis indicates the frequency of detected pathways. (b) the histogram of the numbers of detected pathways for each compound, where the holizontal axis indicates the number of detected pathways for each compound and the vertical axis indicates the frequency of compounds. Red bars indicate the numbers of activated pathways, identified using upregulated genes, and green bars indicate the numbers of inactivated pathways, identified using downregulated genes.

Supplementary Figure S5 Distribution of drug classifications according to the biological pathways that they activate (top) and inactivate (bottom) based on REACTOME. The dendrogram shows the result of clustering pathways according to their similarities of the drug classifications. The fraction of drugs in a

particular classification that affect each pathway is represented by the intensity of color in the appropriate box. The intensity of color indicates the relative frequency (the compound frequency was divided by the number of compounds in each pathway). The boxes are arranged according to the first level of the Anatomical Therapeutic Chemical classification system (ATC code). Drug are assigned the following ATC codes: code A: alimentary tract and metabolism; code B: blood and blood-forming organs; code C: cardiovascular system; code D: dermatologicals; code G: genitourinary system and sex hormones; code H: systemic hormonal preparations, excluding sex hormones and insulins; code J: anti-infectives for systemic use; code L: anti-neoplastic and immunomodulating agents; code M: musculo-skeletal system; code N: nervous system; code P: anti-parasitic products, insecticides and repellents; code R: respiratory system; code S: sensory organs; and code V: various.

Supplementary Figure S6 Distribution of drug classifications according to the biological pathways that they activate (top) and inactivate (bottom) based on REACTOME. The fraction of drugs in a particular classification that affect each pathway is represented by the intensity of color in the appropriate box. The intensity of color indicates the relative frequency (the compound frequency was divided by the number of compounds in each Anatomical Therapeutic Chemical classification system (ATC code)). The boxes are arranged according to the first level of the ATC code. Drug are assigned the following ATC codes: code A: alimentary tract and metabolism; code B: blood and blood-forming organs; code C: cardiovascular system; code D: dermatologicals; code G: genitourinary system and sex hormones; code H: systemic hormonal preparations, excluding sex hormones and insulins; code J: anti-infectives for systemic use; code L: anti-neoplastic and immunomodulating agents; code M: musculo-skeletal system; code N: nervous system; code P: anti-parasitic products, insecticides and repellents; code R: respiratory system; code S: sensory organs; and code V: various.

Supplementary Figure S7 Venn diagram of new compound–protein interactions predicted using CMap and LINCS. The left panel shows the result for the same cell line-matching strategy, and the right panel shows that for the all cell line-matching strategy.

Supplementary Figure S8 Venn diagram of new compound–disease associations predicted using CMap and LINCS. The left panel shows the result for the same cell line-matching strategy, and the right panel shows that for the all cell line-matching strategy.

Supplementary Figure S9 Examples of newly predicted target proteins of drugs (a–c) and pathway enrichment analysis (d). Blue circles denote drugs, red rectangles denote proteins, gray diamonds indicate ATC codes, and gray edges

and red dotted lines denote known interactions and newly predicted interactions, respectively.

Supplementary Figure S10 Dose response curves of four tested drugs in the AR-binding assay run in (a) agonist and (b) antagonist modes. The horizontal axis represents the drug concentrations on a logarithmic scale, and the vertical axis represents the percentages of drug activity. The open circles represent the data points from triplicate experiments.

Supplementary Figure S11 Histograms for evaluating the similarity between gene expression signatures in CMap and LINCS. The left and right panels show the distributions of similarity scores between signatures obtained using cell lines MCF7 and PC3, respectively. The signatures were obtained using the "Biological control" from profiles measured at 6 (6.4) h. The numbers of compounds used to treat both MCF7 and PC3 cells in CMap and LINCS were 331 and 283, respectively. The similarity scores were calculated using the Pearson correlation coefficient.

Supplementary Table S1 Detailed evaluation of target protein prediction using common data.

		6 (6.4) h									24 (24.4) h		
		CMap - Biological control						LINCS - Level 3			LINCS - Level 3		
		signature : all genes			signature : L1000 genes			signature : L1000 genes			signature : L1000 genes		
similarity search strategy	top to bottom gene selection	AUC FPR = 0.1	AUC FPR = 1.0	AUPR	AUC FPR = 0.1	AUC FPR = 1.0	AUPR	AUC FPR = 0.1	AUC FPR = 1.0	AUPR	AUC FPR = 0.1	AUC FPR = 1.0	AUPR
same cell line-matching	top50*	0.0444	0.8356	0.1277	0.0453	0.8329	0.1257	0.0434	0.8244	0.1211	0.0430	0.8207	0.1190
	top100**	0.0439	0.8346	0.1179	0.0449	0.8334	0.1253	0.0425	0.8212	0.1130	0.0425	0.8182	0.1144
	all***	0.0436	0.8288	0.1213	0.0451	0.8310	0.1279	0.0423	0.8203	0.1145	0.0428	0.8175	0.1183
different cell line-matching	top50	0.0429	0.8207	0.1148	0.0424	0.8181	0.1124	0.0395	0.8147	0.1032	0.0406	0.8090	0.1065
	top100	0.0427	0.8221	0.1140	0.0434	0.8239	0.1138	0.0386	0.8130	0.0999	0.0398	0.8075	0.1057
	all	0.0404	0.8178	0.1027	0.0432	0.8283	0.1161	0.0383	0.8099	0.0922	0.0382	0.8063	0.1008
all cell line-matching	top50	0.0446	0.8295	0.1276	0.0453	0.8282	0.1241	0.0431	0.8215	0.1189	0.0430	0.8137	0.1165
	top100	0.0444	0.8287	0.1173	0.0451	0.8315	0.1260	0.0422	0.8198	0.1122	0.0426	0.8122	0.1130
	all	0.0436	0.8225	0.1201	0.0452	0.8279	0.1286	0.0417	0.8169	0.1121	0.0424	0.8106	0.1175
		CMap - Mean centering						LINCS - Level 4			LINCS - Level 4		
		signature : all genes			signature : L1000 genes			signature : L1000 genes			signature : L1000 genes		
similarity search strategy	top to bottom gene selection	AUC FPR = 0.1	AUC FPR = 1.0	AUPR	AUC FPR = 0.1	AUC FPR = 1.0	AUPR	AUC FPR = 0.1	AUC FPR = 1.0	AUPR	AUC FPR = 0.1	AUC FPR = 1.0	AUPR
same cell line-matching	top50	0.0444	0.8366	0.1215	0.0448	0.8286	0.1227	0.0422	0.8158	0.1123	0.0445	0.8275	0.1225
	top100	0.0446	0.8405	0.1234	0.0451	0.8313	0.1244	0.0425	0.8153	0.1132	0.0447	0.8282	0.1194
	all	0.0420	0.8261	0.1228	0.0440	0.8257	0.1248	0.0425	0.8145	0.1115	0.0442	0.8281	0.1205
different cell line-matching	top50	0.0420	0.8226	0.1111	0.0437	0.8281	0.1190	0.0396	0.8103	0.1010	0.0404	0.8126	0.1082
	top100	0.0418	0.8242	0.1106	0.0428	0.8271	0.1131	0.0396	0.8110	0.1012	0.0406	0.8120	0.1100
	all	0.0401	0.8176	0.1036	0.0431	0.8224	0.1179	0.0383	0.8077	0.0992	0.0413	0.8124	0.1100
all cell line-matching	top50	0.0440	0.8295	0.1189	0.0445	0.8278	0.1211	0.0425	0.8162	0.1119	0.0441	0.8238	0.1201
	top100	0.0441	0.8329	0.1212	0.0449	0.8280	0.1223	0.0421	0.8149	0.1108	0.0440	0.8239	0.1155
	all	0.0424	0.8211	0.1230	0.0440	0.8226	0.1257	0.0418	0.8133	0.1065	0.0435	0.8243	0.1167

* The expression values for the top-ranked and bottom-ranked 50 genes were used.

** The expression values for the top-ranked and bottom-ranked 100 genes were used.

*** The expression values for all genes were used.

Supplementary Table S2 Detailed evaluation of target protein prediction using merged data.

		6 (6.4) h									24 (24.4) h		
		CMap - Biological control						LINCS - Level 3			LINCS - Level 3		
		signature : all genes			signature : L1000 genes			signature : L1000 genes			signature : L1000 genes		
similarity search strategy	top to bottom gene selection	AUC FPR = 0.1	AUC FPR = 1.0	AUPR	AUC FPR = 0.1	AUC FPR = 1.0	AUPR	AUC FPR = 0.1	AUC FPR = 1.0	AUPR	AUC FPR = 0.1	AUC FPR = 1.0	AUPR
same cell line-matching	top50*	0.0135	0.5662	0.0241	0.0136	0.5661	0.0236	0.0515	0.8652	0.1021	0.0515	0.8628	0.1015
	top100**	0.0135	0.5661	0.0232	0.0136	0.5661	0.0234	0.0509	0.8635	0.0990	0.0512	0.8618	0.1001
	all***	0.0134	0.5648	0.0232	0.0135	0.5650	0.0241	0.0498	0.8608	0.0985	0.0505	0.8589	0.1024
different cell line-matching	top50	0.0132	0.5656	0.0209	0.0131	0.5653	0.0201	0.0395	0.8288	0.0555	0.0385	0.8247	0.0529
	top100	0.0133	0.5658	0.0212	0.0132	0.5657	0.0206	0.0391	0.8249	0.0547	0.0379	0.8206	0.0521
	all	0.0129	0.5640	0.0206	0.0132	0.5649	0.0228	0.0376	0.8195	0.0505	0.0375	0.8168	0.0501
all cell line-matching	top50	0.0135	0.5664	0.0242	0.0136	0.5662	0.0234	0.0502	0.8600	0.0976	0.0504	0.8589	0.0961
	top100	0.0135	0.5664	0.0235	0.0136	0.5665	0.0234	0.0499	0.8588	0.0963	0.0503	0.8576	0.0964
	all	0.0134	0.5653	0.0229	0.0136	0.5656	0.0242	0.0487	0.8543	0.0955	0.0494	0.8536	0.0980
		CMap - Mean centering						LINCS - Level 4			LINCS - Level 4		
		signature : all genes			signature : L1000 genes			signature : L1000 genes			signature : L1000 genes		
similarity search strategy	top to bottom gene selection	AUC FPR = 0.1	AUC FPR = 1.0	AUPR	AUC FPR = 0.1	AUC FPR = 1.0	AUPR	AUC FPR = 0.1	AUC FPR = 1.0	AUPR	AUC FPR = 0.1	AUC FPR = 1.0	AUPR
same cell line-matching	top50	0.0135	0.5664	0.0235	0.0134	0.5657	0.0231	0.0469	0.8542	0.0767	0.0469	0.8518	0.0820
	top100	0.0134	0.5665	0.0233	0.0134	0.5660	0.0233	0.0469	0.8537	0.0781	0.0477	0.8530	0.0860
	all	0.0132	0.5643	0.0235	0.0133	0.5643	0.0231	0.0468	0.8517	0.0819	0.0470	0.8499	0.0890
different cell line-matching	top50	0.0130	0.5657	0.0206	0.0130	0.5650	0.0209	0.0381	0.8219	0.0501	0.0379	0.8255	0.0510
	top100	0.0130	0.5658	0.0203	0.0130	0.5652	0.0200	0.0388	0.8248	0.0509	0.0386	0.8257	0.0528
	all	0.0128	0.5641	0.0206	0.0131	0.5645	0.0215	0.0393	0.8245	0.0537	0.0387	0.8242	0.0559
all cell line-matching	top50	0.0134	0.5663	0.0233	0.0133	0.5657	0.0229	0.0435	0.8389	0.0682	0.0442	0.8429	0.0726
	top100	0.0134	0.5665	0.0233	0.0133	0.5658	0.0228	0.0447	0.8427	0.0733	0.0453	0.8456	0.0799
	all	0.0131	0.5648	0.0228	0.0133	0.5649	0.0232	0.0455	0.8443	0.0763	0.0457	0.8447	0.0823

* The expression values for the top-ranked and bottom-ranked 50 genes were used.
** The expression values for the top-ranked and bottom-ranked 100 genes were used.
*** The expression values for all genes were used.

Supplementary Table S3 Evaluation of target protein prediction using common data (a) and merged data (b).

a. common data

	LINCS - Level 3 signature : L1000 genes	
similarity search strategy	AUC	AUPR
same tissue-matching	0.8226	0.1225
different tissue-matching	0.8197	0.0973
all tissue-matching	0.8241	0.1192

b. merged data

	LINCS - Level 3 signature : L1000 genes	
similarity search strategy	AUC	AUPR
same tissue-matching	0.8633	0.1025
different tissue-matching	0.8374	0.0574
all tissue-matching	0.8608	0.0998

Supplementary Table S4 The distribution of drugs repositioned from the original disease class to other disease classes using the CMap-based method.

ICD chapter (# of drugs)	I	II	III	IV	V	VI	VII	VIII	IX	X	XI	XII	XIII	XIV	XV	XVI	XVII	XVIII	XIX	XX	XXI	XXII
chapter I (33)	20	11	5	9	3	15	7	0	7	15	3	6	11	2	0	4	0	0	0	0	0	0
chapter II (10)	4	4	1	3	1	2	1	0	1	2	0	0	2	0	0	2	0	0	0	0	0	0
chapter III (2)	2	1	0	1	0	2	1	0	1	1	0	0	0	0	0	0	0	0	0	0	0	0
chapter IV (11)	8	6	3	6	0	6	5	0	2	6	1	2	6	1	0	1	0	0	0	0	0	0
chapter V (2)	1	1	0	0	0	0	0	0	0	0	0	0	1	0	0	0	0	0	0	0	0	0
chapter VI (17)	12	6	5	8	0	11	6	0	5	11	3	4	11	2	0	3	0	0	0	0	0	0
chapter VII (4)	3	1	1	2	0	3	0	0	1	3	1	1	2	1	0	1	0	0	0	0	0	0
chapter VIII (0)	0	0	0	0	0	0	0	0	0	0	0	0	0	0	0	0	0	0	0	0	0	0
chapter IX (5)	3	1	1	2	0	3	1	0	0	3	2	1	3	1	0	3	0	0	0	0	0	0
chapter X (16)	11	4	3	5	0	8	5	0	4	8	2	5	5	1	0	2	0	0	0	0	0	0
chapter XI (3)	2	0	0	1	0	2	1	0	2	2	2	1	2	0	0	1	0	0	0	0	0	0
chapter XII (3)	3	0	0	1	0	2	1	0	1	3	1	0	1	1	0	1	0	0	0	0	0	0
chapter XIII (13)	8	4	1	5	1	9	3	0	3	7	2	2	5	1	0	1	0	0	0	0	0	0
chapter XIV (1)	1	0	0	1	0	1	1	0	1	1	0	1	1	0	0	0	0	0	0	0	0	0
chapter XV (0)	0	0	0	0	0	0	0	0	0	0	0	0	0	0	0	0	0	0	0	0	0	0
chapter XVI (6)	3	2	1	2	0	4	2	0	2	4	1	1	2	0	0	0	0	0	0	0	0	0
chapter XVII (0)	0	0	0	0	0	0	0	0	0	0	0	0	0	0	0	0	0	0	0	0	0	0
chapter XVIII (0)	0	0	0	0	0	0	0	0	0	0	0	0	0	0	0	0	0	0	0	0	0	0
chapter XIX (0)	0	0	0	0	0	0	0	0	0	0	0	0	0	0	0	0	0	0	0	0	0	0
chapter XX (0)	0	0	0	0	0	0	0	0	0	0	0	0	0	0	0	0	0	0	0	0	0	0
chapter XXI (0)	0	0	0	0	0	0	0	0	0	0	0	0	0	0	0	0	0	0	0	0	0	0
chapter XXII (0)	0	0	0	0	0	0	0	0	0	0	0	0	0	0	0	0	0	0	0	0	0	0

Each element represents the number of drugs repositioned from the original disease class to new disease classes using the CMap-based method. The rows indicate the original ICD disease chapters, and the columns indicate the newly predicted ICD disease chapters. Chapter I: certain infectious and parasitic diseases (A00–B99); chapter II: neoplasms (C00–D48); chapter III: diseases of the blood, blood-forming organs, and certain disorders involving the immune mechanism (D50–D89); chapter IV: endocrine, nutritional, and metabolic diseases (E00–E90); chapter V: mental and behavioral disorders (F00–F99); chapter VI: diseases of the nervous system (G00–G99); chapter VII: diseases of the eye and adnexa (H00–H59); chapter VIII: diseases of the ear and mastoid process (H60–H95); chapter IX: diseases of the circulatory system (I00–I99); chapter X: diseases of the respiratory system (J00–J99); chapter XI: diseases of the digestive system (K00–K93); chapter XII: diseases of the skin and subcutaneous tissue (L00–L99); chapter XIII: diseases of the musculoskeletal system and connective tissue (M00–M99); chapter XIV: diseases of the genitourinary system (N00–N99); chapter XV: pregnancy, childbirth, and the puerperium (O00–O99); chapter XVI: certain conditions originating in the perinatal period (P00–P96); chapter XVII: congenital malformations, deformations; and chromosomal abnormalities (Q00–Q99); chapter XVIII: symptoms, signs, and abnormal clinical and laboratory findings not elsewhere classified (R00–R99); chapter XIX: injury, poisoning, and certain other consequences of external causes (S00–T98); chapter XX: external causes of morbidity and mortality (V01–Y98); chapter XXI: factors influencing health status and contact with health services (Z00–Z99); and chapter XXII: codes for special purposes (U00–U99).

Supplementary Table S5 The distribution of drugs repositioned from the original disease class to other disease classes using the LINCS-based method.

ICD chapter (# of drugs)	I	II	III	IV	V	VI	VII	VIII	IX	X	XI	XII	XIII	XIV	XV	XVI	XVII	XVIII	XIX	XX	XXI	XXII
chapter I (103)	88	85	52	85	5	78	37	0	57	84	34	51	72	35	0	42	9	19	9	0	0	15
chapter II (63)	51	51	27	45	12	40	21	0	28	44	23	22	40	9	0	16	13	19	2	0	0	6
chapter III (12)	11	7	2	11	2	9	5	0	7	8	5	7	6	2	0	3	2	2	1	0	0	3
chapter IV (44)	38	34	23	37	8	32	21	0	24	36	20	20	29	10	0	16	11	12	5	0	0	6
chapter V (2)	2	2	1	1	0	2	1	0	1	2	1	1	2	0	0	1	1	1	1	0	0	0
chapter VI (41)	34	29	15	32	3	28	12	0	22	32	18	18	22	10	0	9	6	4	3	0	0	4
chapter VII (8)	7	7	4	8	1	6	0	0	4	6	3	4	6	1	0	2	2	1	1	0	0	1
chapter VIII (0)	0	0	0	0	0	0	0	0	0	0	0	0	0	0	0	0	0	0	0	0	0	0
chapter IX (12)	11	10	5	10	3	8	5	0	5	10	4	7	8	2	0	3	3	3	2	0	0	2
chapter X (52)	44	40	21	43	5	34	17	0	23	30	23	24	28	11	0	13	5	9	3	0	0	6
chapter XI (9)	6	5	4	7	2	6	3	0	2	6	0	4	4	2	0	2	2	1	0	0	0	0
chapter XII (23)	18	16	10	17	1	10	6	0	9	7	4	9	8	1	0	4	0	2	1	0	0	1
chapter XIII (39)	29	23	11	32	2	24	8	0	18	23	10	18	14	5	0	8	2	5	2	0	0	4
chapter XIV (4)	3	2	1	4	0	4	1	0	3	2	2	1	2	0	0	0	0	1	0	0	0	1
chapter XV (0)	0	0	0	0	0	0	0	0	0	0	0	0	0	0	0	0	0	0	0	0	0	0
chapter XVI (12)	10	8	3	10	1	9	2	0	8	9	4	7	7	2	0	0	0	3	3	0	0	1
chapter XVII (3)	3	3	3	3	2	3	1	0	2	2	1	0	2	0	0	0	2	2	0	0	0	0
chapter XVIII (4)	4	3	2	4	2	3	1	0	2	3	2	2	1	1	0	1	2	0	0	0	0	0
chapter XIX (6)	4	2	0	5	1	1	1	0	3	2	0	2	1	0	0	1	0	0	0	0	0	1
chapter XX (0)	0	0	0	0	0	0	0	0	0	0	0	0	0	0	0	0	0	0	0	0	0	0
chapter XXI (0)	0	0	0	0	0	0	0	0	0	0	0	0	0	0	0	0	0	0	0	0	0	0
chapter XXII (2)	2	2	2	2	0	2	1	0	2	2	0	1	1	1	0	1	0	0	1	0	0	0

Each element represents the number of drugs repositioned from the original disease class to new disease classes using the LINCS-based method. The rows indicate the original ICD disease chapters, and the columns indicate the newly predicted ICD disease chapters. Chapter I: certain infectious and parasitic diseases (A00–B99); chapter II: neoplasms (C00–D48); chapter III: diseases of the blood, blood-forming organs, and certain disorders involving the immune mechanism (D50–D89); chapter IV: endocrine, nutritional, and metabolic diseases (E00–E90); chapter V: mental and behavioral disorders (F00–F99); chapter VI: diseases of the nervous system (G00–G99); chapter VII: diseases of the eye and adnexa (H00–H59); chapter VIII: diseases of the ear and mastoid process (H60–H95); chapter IX: diseases of the circulatory system (I00–I99); chapter X: diseases of the respiratory system (J00–J99); chapter XI: diseases of the digestive system (K00–K93); chapter XII: diseases of the skin and subcutaneous tissue (L00–L99); chapter XIII: diseases of the musculoskeletal system and connective tissue (M00–M99); chapter XIV: diseases of the genitourinary system (N00–N99); chapter XV: pregnancy, childbirth, and the puerperium (O00–O99); chapter XVI: certain conditions originating in the perinatal period (P00–P96); chapter XVII: congenital malformations, deformations; and chromosomal abnormalities (Q00–Q99); chapter XVIII: symptoms, signs, and abnormal clinical and laboratory findings not elsewhere classified (R00–R99); chapter XIX: injury, poisoning, and certain other consequences of external causes (S00–T98); chapter XX: external causes of morbidity and mortality (V01–Y98); chapter XXI: factors influencing health status and contact with health services (Z00–Z99); and chapter XXII: codes for special purposes (U00–U99).

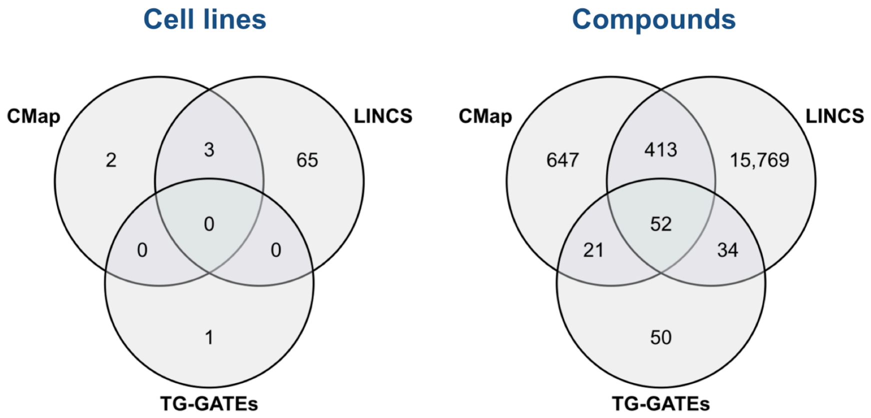

Supplementary Figure S1 Venn diagram of cell lines (left) and chemical compounds (right) present in TG-GATEs, CMap, and LINCS; the three databases containing chemically-induced gene expression profiles.

drug name	ATC code (First level)	cell line	L1000 genes on the cell cycle pathway																																						
			ABL1	BUB1B	CCNA1	CCNA2	CCNB1	CCNB2	CCND1	CCND3	CCNE2	CCNH	CDC20	CDC25A	CDC25B	CDC45	CDK1	CDK2	CDK4	CDK6	CDK7	CDKN1A	CDKN1B	CDKN2A	CHEK1	CHEK2	E2F2	GADD45A	GADD45B	HDAC2	MCM3	MYC	ORC1	PCNA	PLK1	RB1	SFN	SKP1	SMAD3	SMC1A	SMC3	TFDP1	TGFB3	TP53
Alvocidib	-	SKBR3				■			■					■							■		■									■			■		■							
Bortezomib		HCT116							■	■			■		■					■			■												■				■					
Cladribine		MCF7		■	■	■	■	■					■							■			■												■									
		VCAP				■		■					■		■		■										■								■									
Crizotinib		MDAMB231							■	■																			■			■												
Dasatinib		BT20							■					■														■	■			■							■					
		MDAMB231							■		■		■																■			■					■		■					
Doxorubicin hydrochloride	L	VCAP	■			■		■	■				■	■	■								■									■			■									
Fludarabine		VCAP				■	■	■				■	■		■		■																	■	■									
Ixazomib		HEC108				■		■	■				■		■		■						■												■				■					
		MCF7				■								■	■																■				■								■	
Lonidamine		A549				■			■				■	■										■			■				■								■					
Palbociclib		BT20								■	■			■		■		■				■			■						■		■	■										
		MCF7								■	■			■		■		■													■			■								■		
Promazine hydrochloride	N	HA1E	■						■		■			■		■				■									■			■		■										
Terfenadine	R	A549									■	■	■					■										■	■				■			■			■	■				
Vidarabine	J, S	VCAP	■		■	■	■	■					■		■																				■					■				

Supplementary Figure S2 Landmark genes of the cell cycle that are partially downregulated by drug perturbations. Information concerning drugs and cell lines are shown in the first three columns. Drugs are categorized according to the ATC code. Names of drugs and genes are given in alphabetical order. Downregulated genes are colored green. Code J: anti-infectives for systemic use; code L: anti-neoplastic and immunomodulating agents; code N: nervous system; code R: respiratory system; and code S: sensory organs.

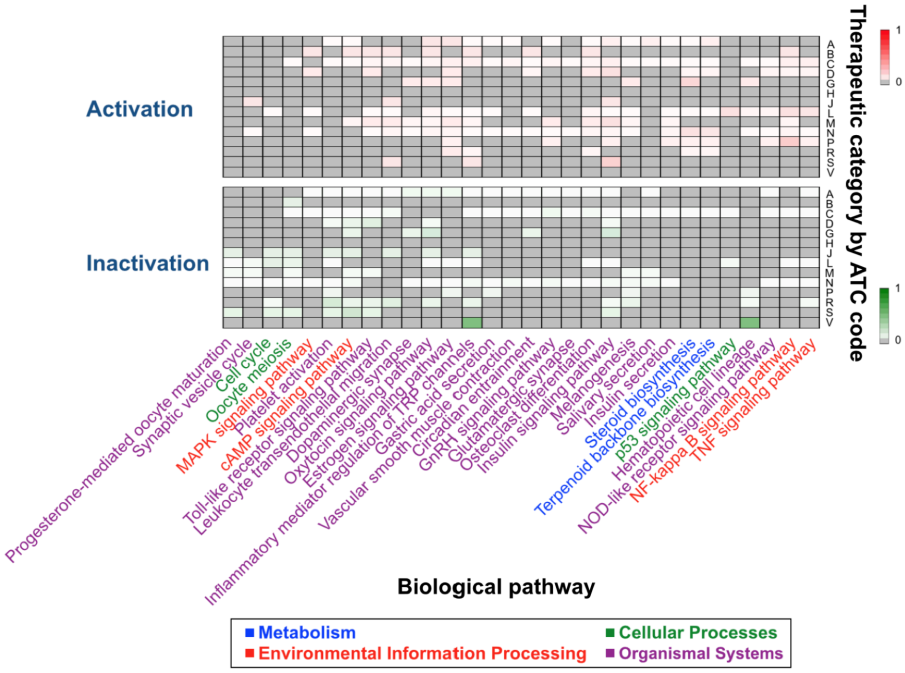

Supplementary Figure S3 Distribution of drug classifications according to the biological pathways that they activate (top) and inactivate (bottom). The fraction of drugs in a particular classification that affect each pathway is represented by the intensity of color in the appropriate box. The intensity of color indicates the relative frequency (the compound frequency was divided by the number of compounds in each Anatomical Therapeutic Chemical classification system (ATC code)). The boxes are arranged according to the first level of the ATC code. Drug are assigned the following ATC codes: code A: alimentary tract and metabolism; code B: blood and blood-forming organs; code C: cardiovascular system; code D: dermatologicals; code G: genitourinary system and sex hormones; code H: systemic hormonal preparations, excluding sex hormones and insulins; code J: anti-infectives for systemic use; code L: anti-neoplastic and immunomodulating agents; code M: musculo-skeletal system; code N: nervous system; code P: anti-parasitic products, insecticides and repellents; code R: respiratory system; code S: sensory organs; and code V: various.

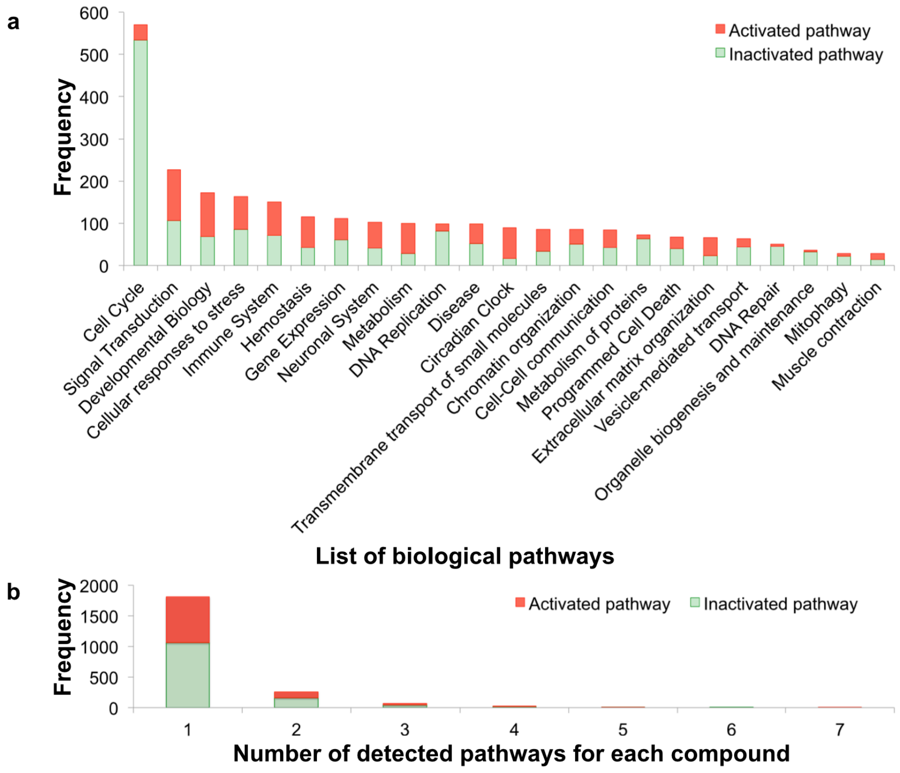

Supplementary Figure S4 Distribution of the identified pathways in REACTOME. (a) the histogram of detected pathways by the result of analyzing all compounds, where the horizontal axis indicates the list of biological pathways and the vertical axis indicates the frequency of detected pathways. (b) the histogram of the numbers of detected pathways for each compound, where the holizontal axis indicates the number of detected pathways for each compound and the vertical axis indicates the frequency of compounds. Red bars indicate the numbers of activated pathways, identified using upregulated genes, and green bars indicate the numbers of inactivated pathways, identified using downregulated genes.

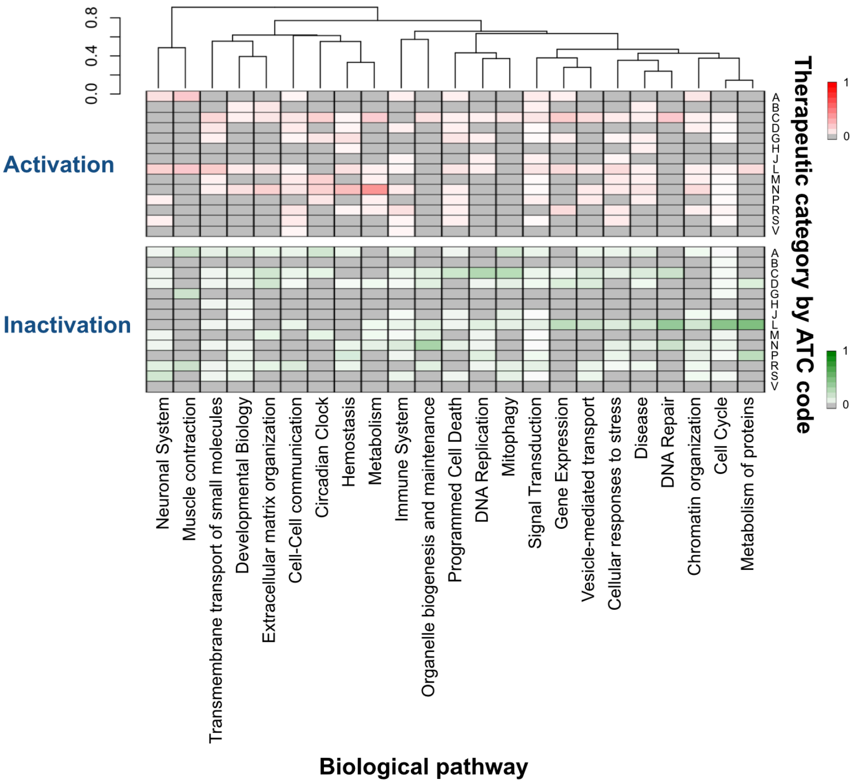

Supplementary Figure S5 Distribution of drug classifications according to the biological pathways that they activate (top) and inactivate (bottom) based on REACTOME. The dendrogram shows the result of clustering pathways according to their similarities of the drug classifications. The fraction of drugs in a particular classification that affect each pathway is represented by the intensity of color in the appropriate box. The intensity of color indicates the relative frequency (the compound frequency was divided by the number of compounds in each pathway). The boxes are arranged according to the first level of the Anatomical Therapeutic Chemical classification system (ATC code). Drug are assigned the following ATC codes: code A: alimentary tract and metabolism; code B: blood and blood-forming organs; code C: cardiovascular system; code D: dermatologicals; code G: genitourinary system and sex hormones; code H: systemic hormonal preparations, excluding sex hormones and insulins; code J: anti-infectives for systemic use; code L: anti-neoplastic and immunomodulating agents; code M: musculo-skeletal system; code N: nervous system; code P: antiparasitic products, insecticides and repellents; code R: respiratory system; code S: sensory organs; and code V: various.

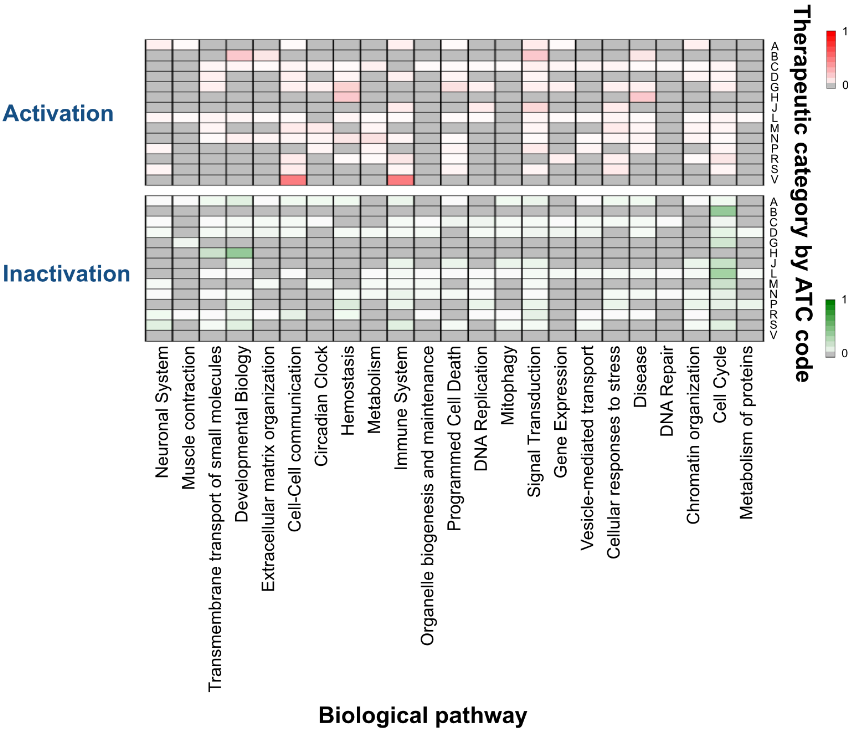

Supplementary Figure S6 Distribution of drug classifications according to the biological pathways that they activate (top) and inactivate (bottom) based on REACTOME. The fraction of drugs in a particular classification that affect each pathway is represented by the intensity of color in the appropriate box. The intensity of color indicates the relative frequency (the compound frequency was divided by the number of compounds in each Anatomical Therapeutic Chemical classification system (ATC code)). The boxes are arranged according to the first level of the ATC code. Drug are assigned the following ATC codes: code A: alimentary tract and metabolism; code B: blood and blood-forming organs; code C: cardiovascular system; code D: dermatologicals; code G: genitourinary system and sex hormones; code H: systemic hormonal preparations, excluding sex hormones and insulins; code J: anti-infectives for systemic use; code L: anti-neoplastic and immunomodulating agents; code M: musculo-skeletal system; code N: nervous system; code P: anti-parasitic products, insecticides and repellents; code R: respiratory system; code S: sensory organs; and code V: various.

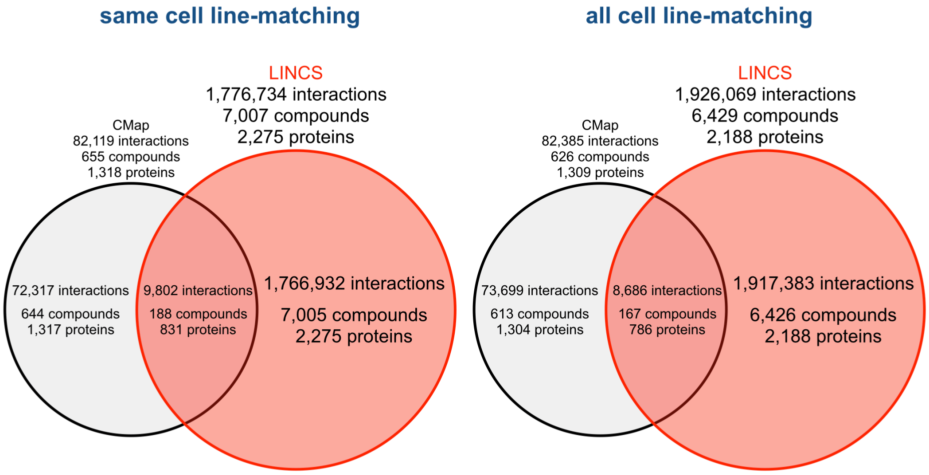

Supplementary Figure S7 Venn diagram of new compound–protein interactions predicted using CMap and LINCS. The left panel shows the result for the same cell line-matching strategy, and the right panel shows that for the all cell line-matching strategy.

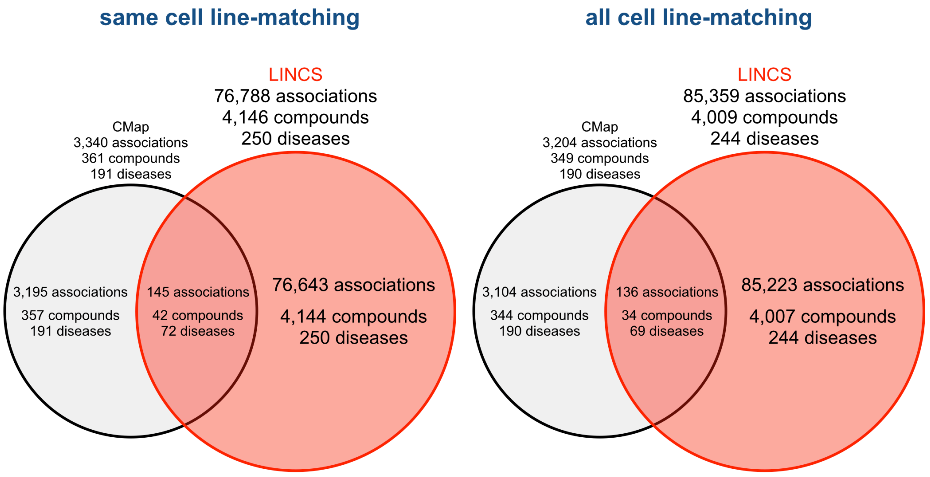

Supplementary Figure S8 Venn diagram of new compound–disease associations predicted using CMap and LINCS. The left panel shows the result for the same cell line-matching strategy, and the right panel shows that for the all cell line-matching strategy.

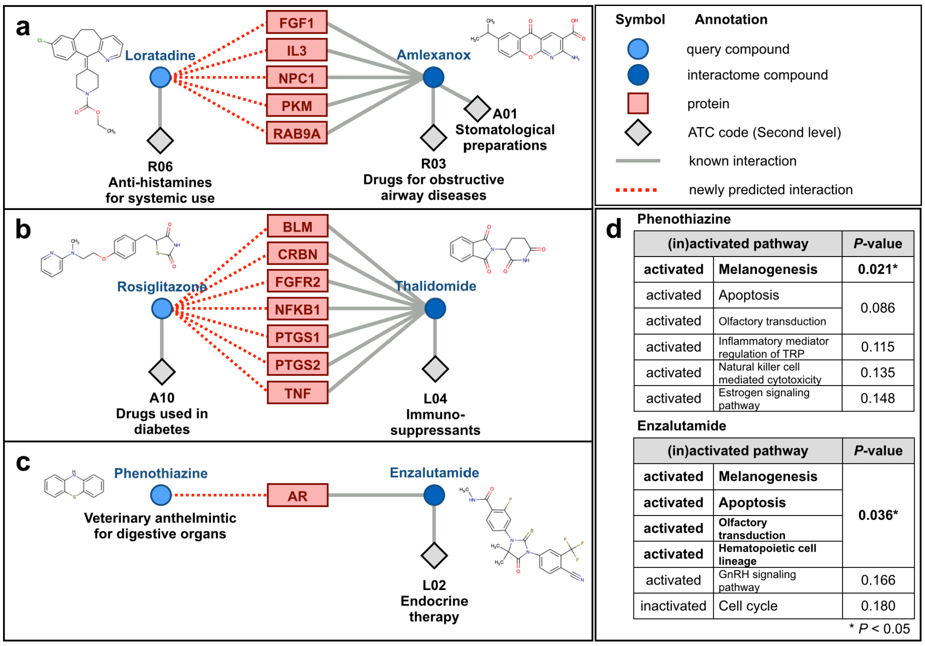

Phenothiazine

(in)activated pathway		*P*-value
**activated**	**Melanogenesis**	**0.021***
activated	Apoptosis	0.086
activated	Olfactory transduction	
activated	Inflammatory mediator regulation of TRP	0.115
activated	Natural killer cell mediated cytotoxicity	0.135
activated	Estrogen signaling pathway	0.148

Enzalutamide

(in)activated pathway		*P*-value
**activated**	**Melanogenesis**	**0.036***
**activated**	**Apoptosis**	
**activated**	**Olfactory transduction**	
**activated**	**Hematopoietic cell lineage**	
activated	GnRH signaling pathway	0.166
inactivated	Cell cycle	0.180

* $P < 0.05$

Supplementary Figure S9 Examples of newly predicted target proteins of drugs (a–c) and pathway enrichment analysis (d). Blue circles denote drugs, red rectangles denote proteins, gray diamonds indicate ATC codes, and gray edges and red dotted lines denote known interactions and newly predicted interactions, respectively.

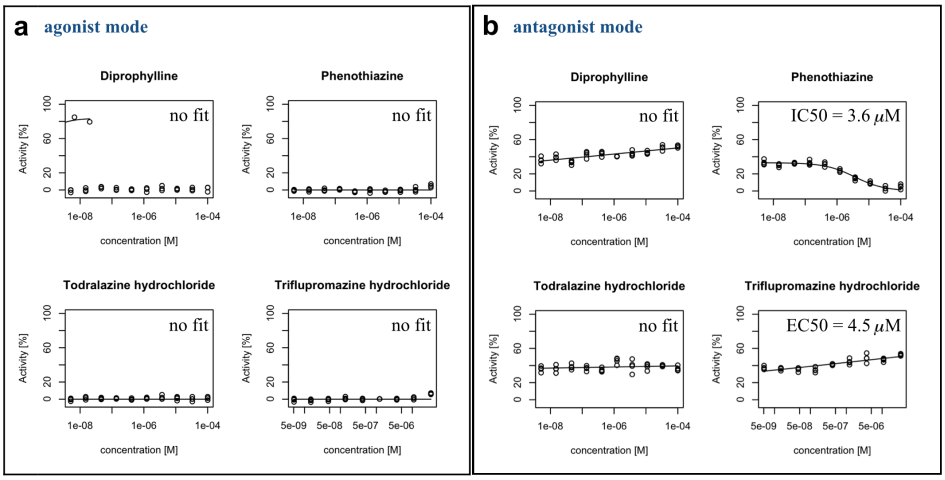

Supplementary Figure S10 Dose response curves of four tested drugs in the AR-binding assay run in (a) agonist and (b) antagonist modes. The horizontal axis represents the drug concentrations on a logarithmic scale, and the vertical axis represents the percentages of drug activity. The open circles represent the data points from triplicate experiments.

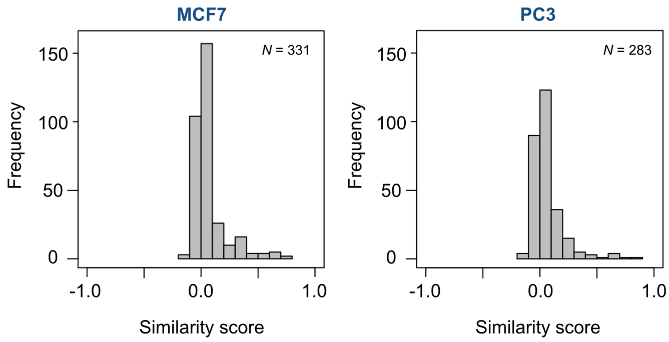

Supplementary Figure S11 Histograms for evaluating the similarity between gene expression signatures in CMap and LINCS. The left and right panels show the distributions of similarity scores between signatures obtained using cell lines MCF7 and PC3, respectively. The signatures were obtained using the "Biological control" from profiles measured at 6 (6.4) h. The numbers of compounds used to treat both MCF7 and PC3 cells in CMap and LINCS were 331 and 283, respectively. The similarity scores were calculated using the Pearson correlation coefficient.
